# Stretchable and Self‐Powered Mechanoluminescent Triboelectric Nanogenerator Fibers toward Wearable Amphibious Electro‐Optical Sensor Textiles

**DOI:** 10.1002/advs.202401109

**Published:** 2024-07-05

**Authors:** Jiajun Wu, Xuhui Zhou, Jie Luo, Jianxian Zhou, Zecheng Lu, Zhiqing Bai, Yuan Fan, Xuedan Chen, Bin Zheng, Zhanyong Wang, Lei Wei, Qichong Zhang

**Affiliations:** ^1^ Key Laboratory of Multifunctional Nanomaterials and Smart Systems Suzhou Institute of Nano‐Tech and Nano‐Bionics Chinese Academy of Sciences Suzhou 215123 China; ^2^ School of Materials Science and Engineering Shanghai Institute of Technology Shanghai 201400 China; ^3^ School of Electrical and Electronic Engineering Nanyang Technological University 50 Nanyang Avenue Singapore 639798 Singapore

**Keywords:** amphibious sensor textile, electro‐optical synergy, mechanoluminescent, noncontact, triboelectric nanogenerator fiber

## Abstract

Flexible electro‐optical dual‐mode sensor fibers with capability of the perceiving and converting mechanical stimuli into digital‐visual signals show good prospects in smart human‐machine interaction interfaces. However, heavy mass, low stretchability, and lack of non‐contact sensing function seriously impede their practical application in wearable electronics. To address these challenges, a stretchable and self‐powered mechanoluminescent triboelectric nanogenerator fiber (MLTENGF) based on lightweight carbon nanotube fiber is successfully constructed. Taking advantage of their mechanoluminescent‐triboelectric synergistic effect, the well‐designed MLTENGF delivers an excellent enhancement electrical signal of 200% and an evident optical signal whether on land or underwater. More encouragingly, the MLTENGF device possesses outstanding stability with almost unchanged sensitivity after stretching for 200%. Furthermore, an extraordinary non‐contact sensing capability with a detection distance of up to 35 cm is achieved for the MLTENGF. As application demonstrations, MLTENGFs can be used for home security monitoring, intelligent zither, traffic vehicle collision avoidance, and underwater communication. Thus, this work accelerates the development of wearable electro‐optical textile electronics for smart human‐machine interaction interfaces.

## Graphical abstract

Stretchable and self‐powered mechanoluminescent triboelectric nanogenerator fiber (MLTENGF) is successfully assembled for smart human‐machine interaction applications. Benefiting from their mechanoluminescent‐triboelectric synergistic effect, the resulting MLTENGF demonstrates prominent electro‐optical signals in response to mechanical stimulus in amphibious environments. Furthermore, such MLTENGF possesses remarkable non‐contact capability with a detection distance of as high as 35 cm.

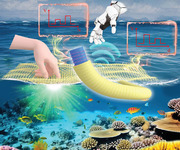

## 1 Introduction

The integration of artificial intelligence (AI) and the Internet of Things (IoT) into wearable electronics has significantly advanced human communication and lifestyles.^[^ ^1^, ^2^, ^3^, ^4^, ^5^, ^6^ ^]^ Applications of fiber electronics (sensors, display, and energy) have accelerated the development of smart homes, personal healthcare, and human‐machine interaction.^[^ ^7^, ^8^, ^9^, ^10^ ^]^ These electro‐optical dual‐mode sensing fibers, capable of perceiving and converting stimuli into digital and analog signals, hold promise for smart human‐machine interaction interfaces.^[^ ^11^, ^12^ ^]^ Persistent concerns about their heavy mass, low stretchability, and lack of non‐contact sensing impact long‐term comfort, durability, and sensitivity, hindering practical application in state‐of‐the‐art smart textiles.^[^ ^13^, ^14^, ^15^ ^]^

To meet stability, comfort, and sensitivity requirements across diverse scenarios, researchers propose a design methodology integrating triboelectric nanogenerator (TENG) and mechanoluminescent materials for dual‐mode electro‐optical sensor fibers.^[^ ^16^, ^17^, ^18^, ^19^ ^]^ The fiber‐shaped TENG efficiently converts mechanical energy into electricity through contact electrification and electrostatic induction, offering advantages such as a broad pressure detection range, superior sensitivity, and material variety.^[^ ^20^, ^21^ ^]^ Carbon materials and platinum‐cured silicone (Ecoflex) emerge as an efficient triboelectric pair in fiber‐based TENGs. Ecoflex, known for its cost‐effectiveness, high electronegativity, unique stretchability (570%), biocompatibility, and flexibility, is a common choice for triboelectric negative materials.^[^ ^22^ ^]^ Carbon materials like carbon nanotubes and graphene serve as excellent electropositive triboelectric materials, with unique mechanical and electrical properties such as robustness, thermodynamic stability, and electrical conductivity.^[^ ^23^ ^]^

For the electro‐optical sensor, prior studies involving the physical combination of separate sensing and display components through active‐matrix circuits present various disadvantages, including complex circuits hindering large‐scale integration.^[^ ^24^, ^25^ ^]^ In contrast, utilizing photon and fluorescence signals as intuitive, efficient, and seamless means of conveying information has shown promise.^[^ ^26^, ^27^ ^]^ Recent research leverages the compatibility between TENG and mechanical luminescence (ML) to achieve real‐time visual sensing output.^[^ ^28^, ^29^ ^]^ ZnS:Cu, a common fluorescent material, provides high‐resolution point luminescence and enhances Ecoflex's electronegativity, improving TENG's electrical output. Although the design methodology of dual‐mode electro‐optical sensor fibers has made great progress, challenges remain in realizing the architecture of “TENG+ML,” especially in the optimal design of materials and structures. Rational microstructure and material design are essential to ensure that ML materials do not compromise sensing sensitivity and monitoring range. Additionally, consistent sensing response capabilities between TENG and ML, without output delay, are critical.

In pursuit of lightweight and versatile sensor fibers supporting both contact and non‐contact fast‐response sensing modes, a coaxial stretchable and self‐powered ML TENG fiber (MLTENGF) is developed. This fiber sandwiches the carbon nanotube fiber (CNTF) electrode between the core layer of stretchable silicone foam bars and an Ecoflex/ZnS:Cu ML encapsulated layer. MLTENGF exhibits outstanding stretchability of exceeding 200% strain and can synergistically export visual and digital signals in response to mechanical stimuli without functional degradation. These mechanically induced responsive mechanisms result from the electric field‐induced charge polarization of the ZnS:Cu fluorescent material during pressure deformation.^[^ ^30^ ^]^ As proof‐of‐concept applications, MLTENGF demonstrates potential in smart homes, personal healthcare, and city scenarios, including physiological motion signal detection, early perception alarm systems, intelligent music platforms, and traffic obstacle avoidance. Special Morse code algorithms reflected by optoelectronic synergies are achieved for underwater rescue through tailored visual‐digital information exchange rules. Particularly, MLTENGF demonstrates a remarkable non‐contact distance of up to 35 cm, signaling a new era of contactless human‐machine interaction without the risk of touching interaction. MLTENGF enables significant application expansion by providing complementary tactile sensing and contacting‐free interaction capabilities, facilitating the further development of fiber electronics in wearable intelligent interaction fields.

## 2 Results and Discussion

**Figure 1:**
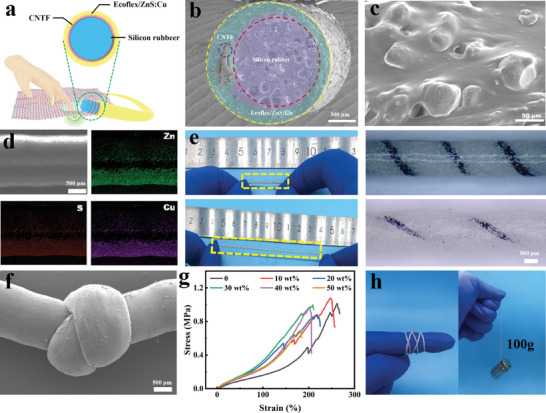
a) Schematic representation of the MLTENGF principle. b) Cross‐sectional SEM image of the MLTENGF. c) SEM image showing the surface morphology of ZnS:Cu particles doped in Ecoflex. d) Energy dispersive spectrum of zinc, sulfur, and copper elements. e) Photo of a stretchable MLTENGF, maintaining good performance when stretched to 200% strain level, alongside its corresponding optical image. f) SEM image of MLTENGF wound into a butterfly‐shaped structure (scale: 500 µm). g) Tensile stress–strain behavior at different doping concentrations (0–50%) with an initial length of 300 mm. h) Photo of a flexible MLTENGF, capable of wrapping around a finger and lifting a 100 g weight.

Cleverly, we introduce an innovative MLTENGF designed to sense and transmit mechanical stimuli. MLTENGF offers feedback in the form of both optical and electrical signals, enabling visual and digital force response. The fabrication of MLTENGF involved a custom process that combines winding and a three‐dimensional coating technique (**Figure** **1**a). Schematic representations of the manufacturing processes and corresponding scanning electron microscopy (SEM) images can be found in Figures S1 and S2 (Supporting Information), respectively. MLTENGF features spiral‐like CNTF assembled in a sandwich configuration, with the core composed of silicon rubber foam circular strips and an outer layer made of a silicone rubber composite (Figure 1b). Cross‐sectional SEM images of MLTENGF reveal a distinct three‐layered structure, with CNTF serving as electrode and the elastic Ecoflex composite as the friction shell. Figure 1c illustrates the mixed‐state nature of ZnS:Cu particles distributed in the Ecoflex polymer matrix,^[^ ^31^ ^]^ further evidenced by visible light exposure under ultraviolet irradiation (Figure S3, Supporting Information). The dense filling of ZnS:Cu is crucial for achieving highly bright devices, and the structural durability of these materials ensures the persistence of ML. Ecoflex, chosen as an effective stress‐transmitting medium due to its strong electron affinity and stability characteristics, prevents the composite material from fracturing during stretching.^[^ ^28^ ^]^ X‐ray diffraction spectra of ZnS:Cu show three major diffraction peaks (Figure S4, Supporting Information) corresponding to crystal planes (111), (220), and (311).^[^ ^31^ ^]^ Additionally, Figure 1d reveals a concentrated distribution of Zn, S, and Cu elements, indicating well‐distributed ZnS:Cu coating, which guarantees a stable light source under mechanical stimuli. As illustrated in Figure 1e, white ZnS:Cu particles remain evenly distributed along the axial direction of the device body even under over 200% strain, with only slight increases in pitch between the black CNTF during stretching, and no significant fractures occur. These observations demonstrate the superior fibrous structure durability and mechanical flexibility attributed to the intrinsically elastic nature of silicon rubber substrates, allowing for robust weaving, as seen in knot‐tying operations (Figure 1f). Consequently, the stretchability of the fiber decreases from 260% to 190% as the ZnS:Cu content increases to 50 wt.%, and the tensile strength decreases from 1.0 to 0.6 MPa (Figure 1g). The progressive distortions observed, particularly after 150% strain, are likely due to the helical structure of CNTF tending to straighten in the linear direction and slight sliding movements caused by the irregular structures of ZnS:Cu particles. The exceptional elastic deformation and stable tensile strength characteristics of MLTENGF enable them to be wrapped around a finger, as presented in Figure 1h, supporting a weight of up to 100 g.

**Figure 2:**
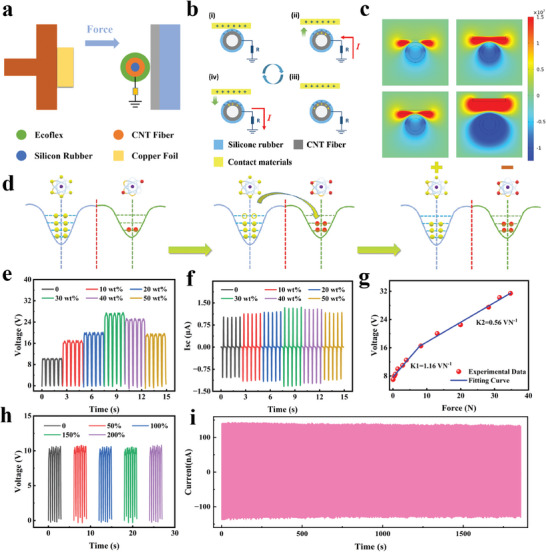
a) Dynamic loading structure schematic for MLTENGF frictional electrical performance. b) Schematic representation of the working principle of the MLTENGF. c) Numerical calculation of the corresponding electric potential distribution using COMSOL software. d) Atomic‐scale electron cloud potential well model describing the charge transfer at the copper foil and Ecoflex/ZnS:Cu contact interface. e) Variation in *V*_OC_ and f) *I*_SC_ at different doping concentrations (0–50%). The output voltage of the MLTENGF under g) different applied loads and i) different stretch levels. i) Stability test of the MLTENGF under a specific stretch level of 200%.

To evaluate the frictional electrical output performance of MLTENGF, encompassing open‐circuit voltage (*V*_oc_), short‐circuit current (*I*_sc_), and transferred charge (*Q*_sc_), a dynamic loading test platform was established for assessing MLTENGF frictional electrical performance (**Figure** **2**a). In the setup, a copper foil was affixed to a barrier, and a 4 cm long MLTENGF was linked to a linear motor, serving as the frictional material. The underlying mechanism of the MLTENGF operation is grounded in the interplay between triboelectric charging and electrostatic induction (Figure 2b). As the copper foil contacts Ecoflex, negative frictional charges accumulate on the Ecoflex surface, inducing positive charges on the copper foil. When the copper foil gradually separates, electrons flow from the CNTF to the ground in a short‐circuit state, inducing positive charges on the electrodes until charge equilibrium is reached. As the frictional material approaches Ecoflex again, reverse electrostatic induction causes electron flow in the opposite direction. The repetitive contact‐separation motion of the linear motor results in the continuous generation of alternating current. For a more quantitative understanding of the power generation process, COMSOL Multiphysics and the electrostatic module were employed to simulate the potential distribution of various components (Figure 2c). When the atoms of both materials are distant, with no overlap between their electron clouds, they maintain their respective orbits. Upon contact, their electron clouds significantly overlap, reducing the interatomic potential barrier, and prompting electrons from higher orbits to transfer to unoccupied orbits. Following the separation of the copper foil and Ecoflex, positive and negative charges remain on their surfaces, respectively (Figure 2d).^[^ ^32^ ^]^ MLTENGF doped with various ZnS:Cu/Ecoflex concentrations (ranging from 0% to 50%) exhibit varying electrical output performances. MLTENGF with a 30% ZnS:Cu/Ecoflex doping ratio exhibits excellent electrical performance, leading to a significant increase in both *V*_OC_ and *I*_SC_ from 10 to 28 V and from 1 to 1.3 µA, respectively (Figure 2e,f). Notably, operational frequency significantly influences the textile‐based TENG output. Under a specific stretch level (200%), within the 1–3 Hz range, *V*_OC_ and *I*_SC_ measurements consistently remain stable (Figure S5a,b, Supporting Information). The sensitivity varies with applied loads, ranging from 0.06 to 34.7 N, with higher sensitivity observed under lower‐pressure conditions (Figure 2g). In order to evaluate the stability of MLTENGF under stretching states from 0% to 200%, we conducted an intermittent impact test on MLTENGF and analyzed the corresponding voltage changes. The results demonstrate the unchanged voltage‐strain sensitivity of MLTENGF, implying its excellent stability (Figure 2h). Under a specific stretch level (200%), the 5000 load tests confirmed MLTENGF durability, with no significant attenuation of ISC after 5000 loading cycles (Figure 2i). SEM images of MLTENGF post‐cycling (Figure S6, Supporting Information) matched those observed before cycling, affirming their robustness. Furthermore, waterproofing and machine washability, crucial requirements for textile applications, were evaluated.^[^ ^17^ ^]^ Figure S7 (Supporting Information) demonstrates the superhydrophilicity of MLTENGF, and Figure S8a,b (Supporting Information) illustrates the washing environment and washing durability tests under a specific stretch level (200%). After multiple washes, there was no significant reduction in the electrical output of MLTENGF. These findings affirm the excellent mechanical robustness and reliability of MLTENGF in practical applications.

**Figure 3:**
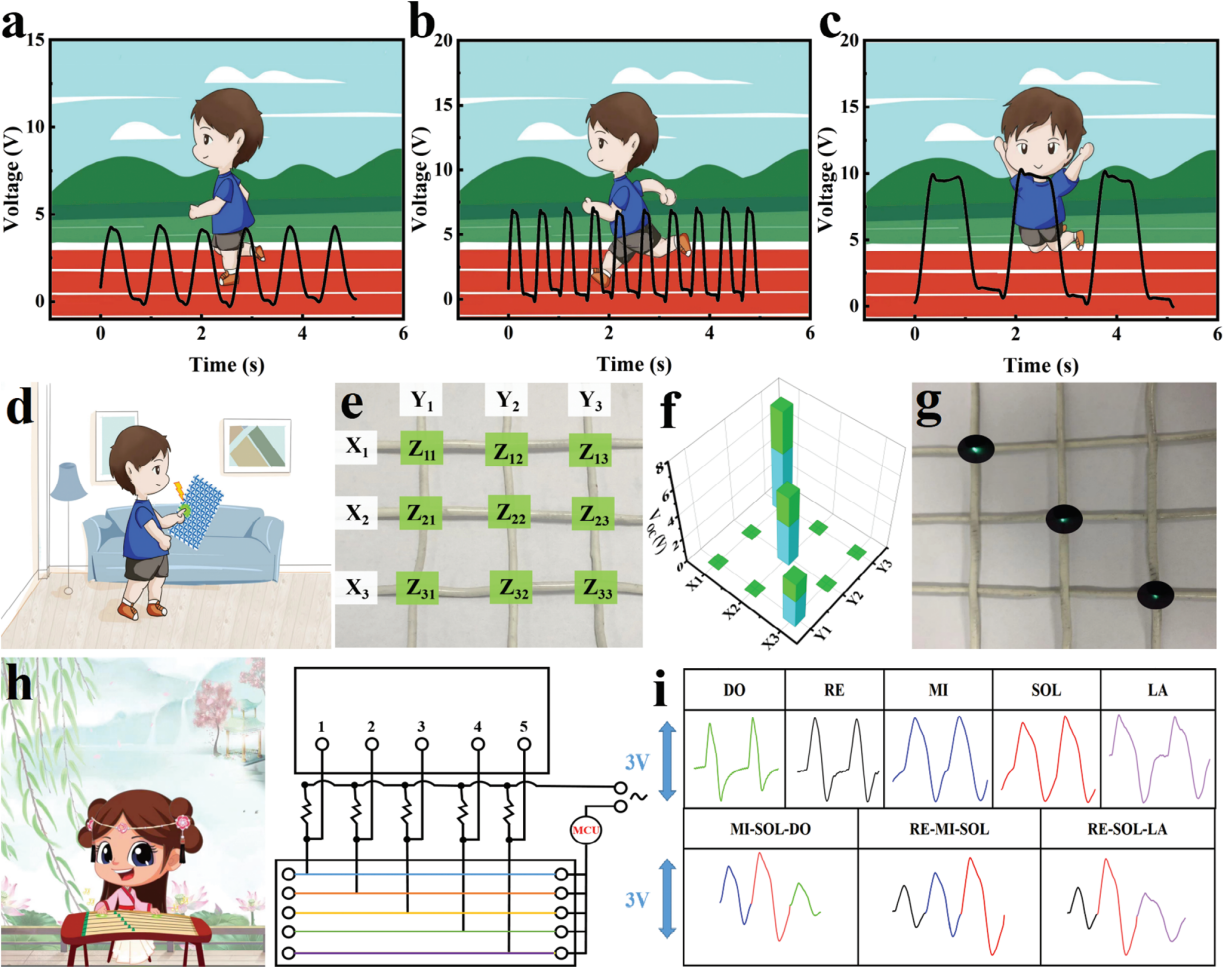
Response of voltage signals to different ranges of motion: a) walking, b) running, and c) jumping. d) Schematic representation of the principle for spatial pressure distribution sensing in TENG under the influence of photoelectric synergy. e) Photo of electronic fabric with a 3 × 3‐pixel MLTENGF sensor. f,g) Output voltage (*V*_OC_) and luminosity of each pixel MLTENGF sensor array's pressure distribution when pressing Z_31_, Z_22_, and Z_13_ with fingers. h) Schematic and equivalent circuit of an “intelligent zither” based on MLTENGF sensors. i) Output signals for five notes (DO, RE, MI, SOL, and LA) and arbitrary note combinations of the “intelligent zither”.

Utilizing the remarkable flexibility, stretchability, and ML of MLTENGF, a self‐powered human motion detection carpet was developed. MLTENGF woven into the carpet generated electrical signals through the periodic contact and separation of a tester's feet as they walked, ran, or jumped on it (**Figure** **3**a–c). The generated electrical power from MLTENGF conversion can be stored in capacitors for future use (Figure S9a–c, Supporting Information). A 1 µF capacitor reaches a 5 V saturation voltage in just 5 s, capable of powering electronic devices like LEDs, displaying the typical character “TENG.” The TENG is versatile in creating breathable, comfortable wearables for contact‐based and non‐contact‐based sensing and monitoring. This technology allows for contact‐based pressure monitoring and non‐contact motion trajectory tracking.^[^ ^33^ ^]^ By weaving MLTENGF fibers in both the warp and weft directions, a pressure sensor array was formed with pixels at the overlapping regions of each warp and weft yarn (Figure 3d). During testing, we monitored the V_OC_ values of all MLTENGFs, each sensor covered by an Ecoflex protective layer to prevent electrical signal interference. In Figure 3e, the yellow column represents the voltage generated in the X direction, and the green column represents the voltage generated in the Y direction. We demonstrated precise recognition of different spatial pressures under visible light, achieved through finger presses, placing doll, and adding weight (200 g) to the pressure sensor array (Figure S10a–c). Figure 3f,g illustrates that individual pressure on Z11, Z22, and Z33 results in output voltages of 3.88, 5.71, and 7.89 V, accompanied by varying green light brightness. The luminescence from MLTENGF pressing is attributed to the stress transfer mechanism of chemically cross‐linked thermosetting elastomer ZnS:Cu/Ecoflex (Figure S11, Supporting Information).^[^ ^34^ ^]^ Remarkably, MLTENGF exhibits high sensitivity even in high‐pressure environments, with varying green light intensity serving as a direct and visible means of pressure perception. TENG technology enables the creation and development of electronic keyboards, exemplified by the “intelligent zither” musical instrument (Figure 3h). A simple touch activates the music, with MLTENGF sensors displaying a quick 70 ms response time and an 80 ms recovery time following finger movement under a specific stretch level (200%) (Figure S12a,b, Supporting Information). Under a specific stretch level (200%), an intelligent music platform, detailed in Figure S13 (Supporting Information), was established based on the circuit. This platform incorporated five MLTENGFs, each corresponding to a different musical note. Data collection involved connecting all five electrodes to a pre‐programmed circuit board controlled by a computer. Sensor interface signals were processed using MATLAB and converted into musical notes. To vividly showcase the smart guzheng application, a supplementary video was prepared (Video S1, Supporting Information). Finger plucking different MLTENGF generated electrical signals, resulting in the play of different musical notes (Figure 3i). The response delay was virtually imperceptible.

**Figure 4:**
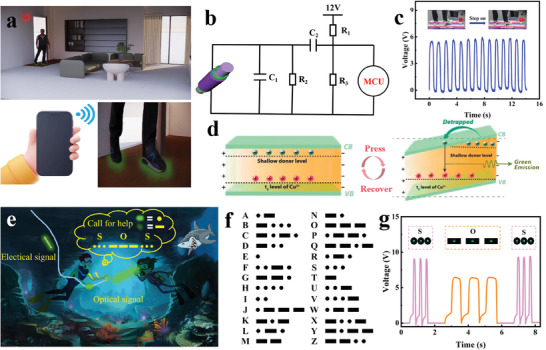
a) Schematic representation of an anti‐theft alarm carpet. b) Equivalent circuit diagram of the anti‐theft alarm carpet. c) Output signals of the anti‐theft alarm carpet in “On–Off” states. d) Schematic energy level diagram for green luminescence in ZnS:Cu under stress. e) Photoelectrically coordinated communication of MLTENGF pressure sensors in an aquatic environment using Morse code. f) Schematic diagram of Morse code principles. g) Transmission of corresponding information underwater by MLTENGF pressure sensors using Morse code.

With precise pressure distribution detection and motion trajectory monitoring capabilities, the TENG textile also finds valuable applications in components of home anti‐theft systems (**Figure** **4**a).^[^ ^35^ ^]^ The theft alarm carpet's equivalent circuit diagram is depicted in Figure 4b. When an intruder enters a home and steps on the carpet, MLTENGF generates an output signal that activates an alarm emitting a green light (Figure 4c). This signal is then transmitted to a mobile phone via Bluetooth wireless communication (Video S2, Supporting Information). Notably, MLTENGF can discern whether the individual is the owner, or an intruder based on their weight (Figure S14, Supporting Information). Figure 4d presents a schematic energy level diagram for the Cu‐doped ZnS semiconductor. Sulfur vacancies within ZnS compounds establish two energy levels: a shallow donor level capturing electrons and a t_2_ acceptor level capturing Cu^2+^ holes. This effect results in the capture of electrons from the valence band to the impurity shallow donor level. Under pressure, these captured electrons are likely to recombine with Cu^2+^ t_2_ acceptors (holes). Consequently, the observed green emission arises from carrier recombination between Cu^2+^ ions in the shallow donor level (sulfur vacancy) and the t_2_ level.^[^ ^36^ ^]^ In the realm of deep‐sea resource exploration, underwater communication technology has garnered interest for its ability to transmit information securely while protecting divers and other underwater personnel. The waterproof qualities of MLTENGF suggest its potential application in underwater rescue operations (Figure 4e).^[^ ^37^ ^]^ Morse code, a well‐established method for transmitting information, comprises “dots” and “dashes” representing distinct Latin letters arranged in specific patterns (Figure 4f).^[^ ^38^ ^]^ Intense light and short‐duration pressing produce variations in photoelectric signals, corresponding to “dots”, while weak light and long‐duration pressing represent “dashes”. Harnessing the photoelectric synergy capabilities of the MLTENGF, introduces an innovative self‐powered underwater solution for rescue, sensing, and information interaction, eliminating the necessity for other integrated electronic components and modules in underwater operations. As illustrated in Figure 4g, “SOS” messages can be effectively transmitted through electro‐optical synergy by rhythmically pressing the MLTENGF and recording different combinations of lines and dots underwater. Diverse light patterns and pressing frequencies convey distinct electrical signals, which are concurrently transmitted to shore‐based companions via the MLTENGF sensors.

**Figure 5:**
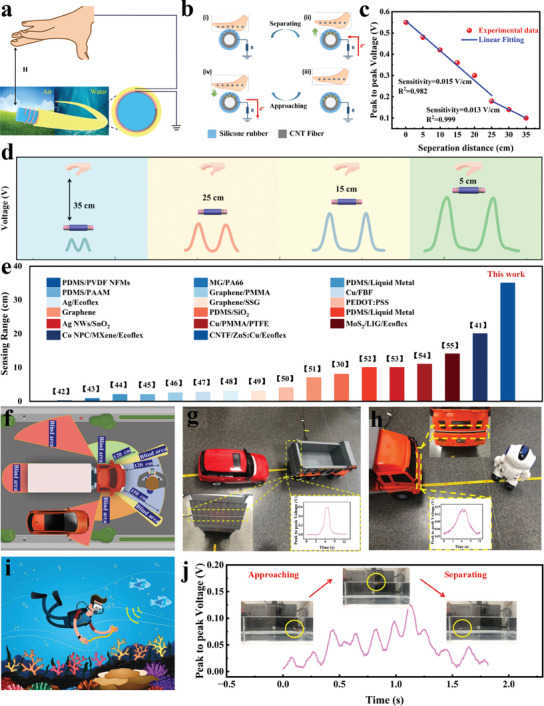
a) Schematic representation of a non‐contact sensing system with Ecoflex/ZnS:Cu as the charge generation layer and CNTF as the charge capture layer. b) Mechanism of non‐contact induction in MLTENGF. c) Variations in output voltage and sensitivity with separation distance. d) Output voltages correspond to different distances. e) Non‐contact sensing performance of the reported proximity sensor. f) Illustration of MLTENGF applications in pedestrian and vehicle collision avoidance. g,h) Application scenarios of obstacle MLTENGF sensors for avoiding vehicles (rear) and robots (front). i) Schematic representation of non‐contact MLTENGF sensors detecting proximity to unknown marine life forms. j) Real‐time output signals of non‐contact MLTENGF sensors based on the approach and retreat of fish in aquatic environments.

Non‐contact TENG can achieve longer‐distance object motion sensing, and because the device is not pressed or worn during the sensing process, its durability and reliability are even better.^[^ ^39^ ^]^ We have developed a non‐contact MLTENGF utilizing the human hand as the positive electrode and Ecoflex/ZnS:Cu as the negative electrode while maintaining excellent waterproof properties (**Figure** **5**a). The ZnS:Cu/Ecoflex layer, serving as the charge generation layer, creates surface charges by forming micro capacitors within the polymer matrix and generating macrodipoles, which enhances the electron affinity properties of ZnS:Cu/Ecoflex. In the MLTENGF operation, it works in a single‐electrode non‐contact mode, with the hand acting as the positive friction layer and ZnS:Cu/Ecoflex as the negative friction layer. Compared to the hand, ZnS:Cu/Ecoflex exhibits a stronger tendency to acquire electrons, making it ideal as the negative friction layer.^[^ ^40^ ^]^ When the hand approaches the negative friction layer, the potential difference between the electrode layer and the ground induces electron flow, generating an electrical signal in CNTF, which serves as the charge capture layer. As the hand starts to move away from the negative friction layer, electrons flow in the opposite direction from the ground to the electrode layer, completing a full cycle of AC signal generation (Figure 5b).

At a separation distance of 0.1 cm, the maximum peak‐to‐peak voltage reaches 0.55 V, and the MLTENGF sensor exhibits two linear regions, labeled as R_1_ (0.1–25 cm) with a sensitivity of 0.015 V cm^−1^ and linear fit of 0.982, and R_2_ (25–35 cm) with a sensitivity of 0.013 V cm^−1^ and linear fit of 0.999. The distance detection threshold ranges from 0.1 to 35 cm, with the voltage decreasing from 0.55 to 0.1 V (Figure 5c). The potential distribution between the ground layer and the electrode layer is visualized through COMSOL simulation during this process, as shown in Figure S15 (Supporting Information). The graph displays voltage variation relative to the distance between the hand and the MLTENGF is depicted (Figure 5d). The MLTENGF demonstrates remarkable sensitivity across a broad range, surpassing 35 cm. This maximum sensing distance outperforms the values in most previously reported literature (Figure 5e).^[^ ^41^, ^42^, ^43^, ^44^, ^45^, ^46^, ^47^, ^48^, ^49^, ^50^, ^51^, ^52^, ^53^, ^54^ ^]^ Non‐contact TENG sensors are pivotal for vehicle obstacle avoidance, especially for large trucks prone to collision accidents due to blind spots (Figure 5f).^[^ ^40^ ^]^ The response and recovery times of non‐contact TENG are critical for effective obstacle avoidance in vehicles. When the object approaches, the MLTENGF responds in 100 ms and recovers in 100 ms (Figure S22a,b). As demonstrated in Figure 5g, the MLTENGF sensor is installed on the rear of a large truck to detect external objects. When an object comes within 30 cm of the truck's rear, MLTENGF sensor promptly detects it and provides real‐time data to an oscilloscope monitor (Video S3, Supporting Information). The MLTENGF demonstrates consistent behavior in underwater settings. The dielectric constant of water, being 64 times that of air, results in higher output voltage in water (Figure S23a, Supporting Information). At a 0.1 cm separation distance, the MLTENGF sensor achieves a maximum peak‐to‐peak voltage of 1.75 V and exhibits two linear regions, denoted as R_1_ and R_2_. The sensitivity is 0.03 V cm^−1^ with a linear fit of 0.991 in the R_1_ region (0.1–25 cm) and the sensitivity is 0.024 V cm^−1^ with a linear fit of 0.999 in the R_2_ region (25–35 cm). The distance detection range spans from 0.1 to 35 cm, resulting in a voltage drop from 1.75 to 0.87 V (Figure S23b, Supporting Information). Consequently, the performance of MLTENGF is closely linked to the separation distance, with improved self‐powered position monitoring as the separation distance decreases. The MLTENGF excellent sealing and sensitivity enable its use in underwater non‐contact detection (Figure 5i). By installing the MLTENGF sensors underwater, we achieve real‐time signal changes based on fish movement, illustrated in Figure 5j and Video S4 (Supporting Information). These demonstrations highlight the MLTENGF suitability for cost‐effective, highly sensitive non‐contact sensing systems. The MLTENGF ensures stable, long‐term output underwater, as demonstrated by the consistent short‐circuit current over sixty days under fixed frequency conditions (Figure S24, Supporting Information).

## 3 Conclusion

In summary, we constructed a stretchable and self‐powered amphibious MLTENGF with the capacity for fast‐response sensing in both contact and non‐contact modes. The newly developed MLTENGF, engineered with a sequential assembly of CNTF and Ecoflex/ZnS:Cu encapsulation materials on stretchable silicone foam cores, exhibits stable functionality under strains of up to 200%, addressing concerns raised earlier about the heavy mass and low stretchability of conventional electro‐optical dual‐mode sensing fibers. Significantly, the MLTENGF excels in non‐contact sensing, reaching up to 35 cm, an advancement toward realizing contactless human‐machine interaction. Demonstrated important applications, including health monitoring, pressure sensing, smart zithers, alarms, and collision avoidance, present the great superiority of smart fiber electronics. Additionally, the MLTENGF's unique underwater sensing, rescue applications, and the specialized Morse code optoelectronic synergy showcase its versatility in addressing imperceptibility, facilitating information exchange, and supporting rescue operations. In essence, the MLTENGF stands as a development of the integration of TENG and ML materials. Its features and diverse applications overcome limitations in current multimode textile electronics, pointing toward potential advancements in future smart human‐machine interaction interfaces.

## 4 Experimental Section

### Preparation of ZnS:Cu/Ecoflex Solution

Initially, ZnS:Cu powder was thoroughly cleaned with n‐hexane (99%, Aladdin, GC) as a dispersant using an ultrasonic cleaner until the solution completely evaporated. Subsequently, components A and B were mixed in a 1:1 mass ratio to prepare a platinum‐catalyzed silicone rubber (Ecoflex 00–30) precursor solution. Finally, ZnS:Cu was incorporated as a doping material into the Ecoflex precursor solution to create the ZnS:Cu/Ecoflex solution, which was continuously stirred at 600 rpm for 30 min.

### Fabrication of Wearable Coaxial‐Fiber MLTENGF Sensor

A flexible and stretchable multilayer mechanical luminescent TENG material was fabricated step by step using winding and three‐dimensional coating techniques with a self‐made apparatus. In detail, a 20G (D_n_ = 0.6 mm) needle was used, and the ZnS:Cu/Ecoflex was coated onto a silicone foam round rod wrapped with CNTF under the following conditions: injection rate (*V*_i_) of 1.0 mm min^−1^, step rate (*V*_s_) of 100 mm min^−1^, and rotation rate (*V*_r_) of 200 rpm. The resulting coaxial yarn was dried in a convection oven at 60 °C for 4 h. Thus, a ZnS:Cu/Ecoflex mechanical luminescent composite TENG coaxial yarn sensor was prepared.

## Conflict of Interest

The authors declare no conflict of interest.

## Supporting information

Supporting Information

Supplemental Video 1

Supplemental Video 2

Supplemental Video 3

Supplemental Video 4

## Data Availability

The data that support the findings of this study are available from the corresponding author upon reasonable request.
