# Supplementary material for: Stretchable and Self‐Powered Mechanoluminescent Triboelectric Nanogenerator Fibers toward Wearable Amphibious Electro‐Optical Sensor Textiles
Source: Adv Sci (Weinh). 2024 Jul 5;11(34):2401109. doi: 10.1002/advs.202401109 (PMC11425994; doi:10.1002/advs.202401109)
Supplement: Supplementary file 1 — Supporting Information [file ADVS-11-2401109-s005.docx]

Supporting Information

Title: **Stretchable and Self-Powered Mechanoluminescent Triboelectric Nanogenerator Fibers toward Wearable Amphibious Electro-Optical Sensor Textiles**

*Jiajun Wu^+^, Xuhui Zhou^+^, Jie Luo^+^, Jianxian Zhou, Zecheng Lu, Zhiqing Bai, Yuan Fan, Xuedan Chen, Bin Zheng, Zhanyong Wang, Lei Wei, Qichong Zhang**

[*] E-mail: [qczhang2016@sinano.ac.cn](mailto:qczhang2016@sinano.ac.cn)

**Materials**

N-Hexane (99%, Aladdin, GC), ZnS:Cu (Shanghai Keyan Phosphor Technology Co.Ltd.), Ecoflex 00-30 (Smooth-On, Inc.).

**Characterization and measurements**

The MLTENGF were morphologically characterized using a scanning electron microscope (SEM) (Hitachi S4800). The mechanical tensile properties of the MLTENGF were tested at a constant speed of 5 mm min^−1^ using an ESM301 tensile tester. Contact angles of the MLTENGF were characterized using a contact angle analyzer (OCA40Micro, Germany). X-ray diffraction (XRD) (D8-ADVANCE) was used to characterize the ZnS:Cu fluorescent powder. Optical images were captured using an optical microscope (BX521, OLYMPUS). The MLTENGF sensors were mounted on an Instron 3342 universal testing machine to test their output voltage under different pressures, with the signals recorded using a Keithley 6514. Three voltage measurements were taken for each pressure, and the average was calculated as the final data. Here, the MLTENGFs were cut into specimens of 4 cm in length and clamped on both sides with fixtures. A linear motor (Linmot E1100) induced periodic contact-separation motion to measure the electrical output capacity of the TENG yarn. To measure the electrical output capacity of the MLTENGF, an external force was applied by the linear motor (Linmot E1100), which provided periodic contact-separation motion. The V_OC_, I_SC_, and Q_SC_ of the MLTENGF were determined using an electrostatic meter (Keithley 6517).

**Simulations**

Use the COMSOL software static module to simulate its electrostatic performance. All AC -drive electrical/electronic devices consume electricity and generate electromagnetic energy when runtime. The intercourse material generated by the adjacent appliances is continuously polarized by the alternating electric field. Here, we consider a system, including four active states: electrical appliances, energy loss layers, air and induction layers. Metal layers and dielectric layers are used to simulate electrical components and energy loss layers. In order to simulate the transmodation field generated, assuming the equipment with positive charge and negative charge on the appliance. Let's set up infinite potential as the ground.

Set the relative dielectric constants of the equipment, energy loss layer, air, and teng conductive composite fiber to 0.0001, 3, 1, and 3.6, respectively. In order to simulate the alternating electric field formed, assuming the positive charge and negative charge density of the surface of the appliance is ± 0.5 C m^−2^. The potential generated by electrical devices can induce static electricity in tee. The influence of the intercourse on the electric field on tee is reduced as the distance between them increases (from 0 to 2mm), so the output is reduced. The alternating electrostatic potential generated in the MLTENGF composite electrode can drive the flow of free electrons inside.


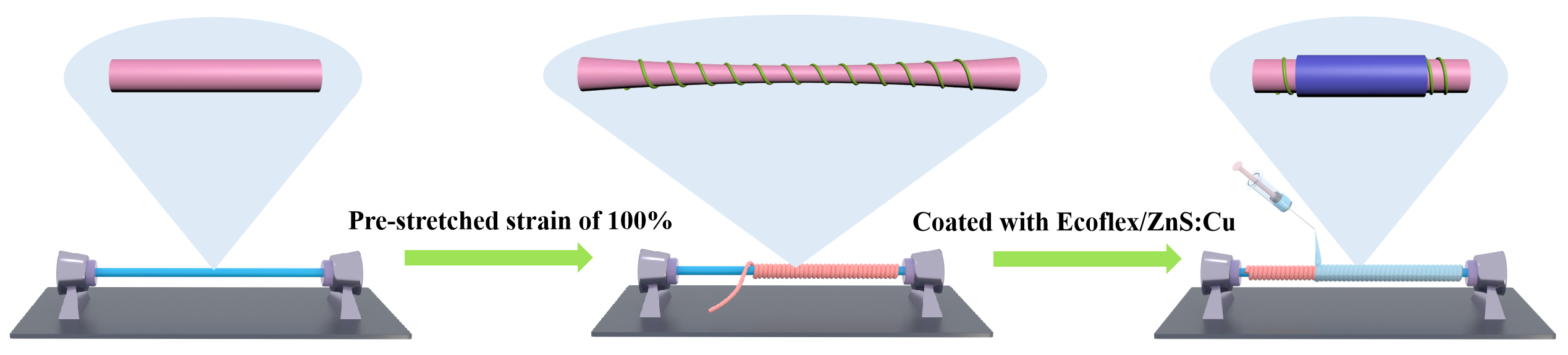


**Figure S1.** The fabrication process of MLTENGF..


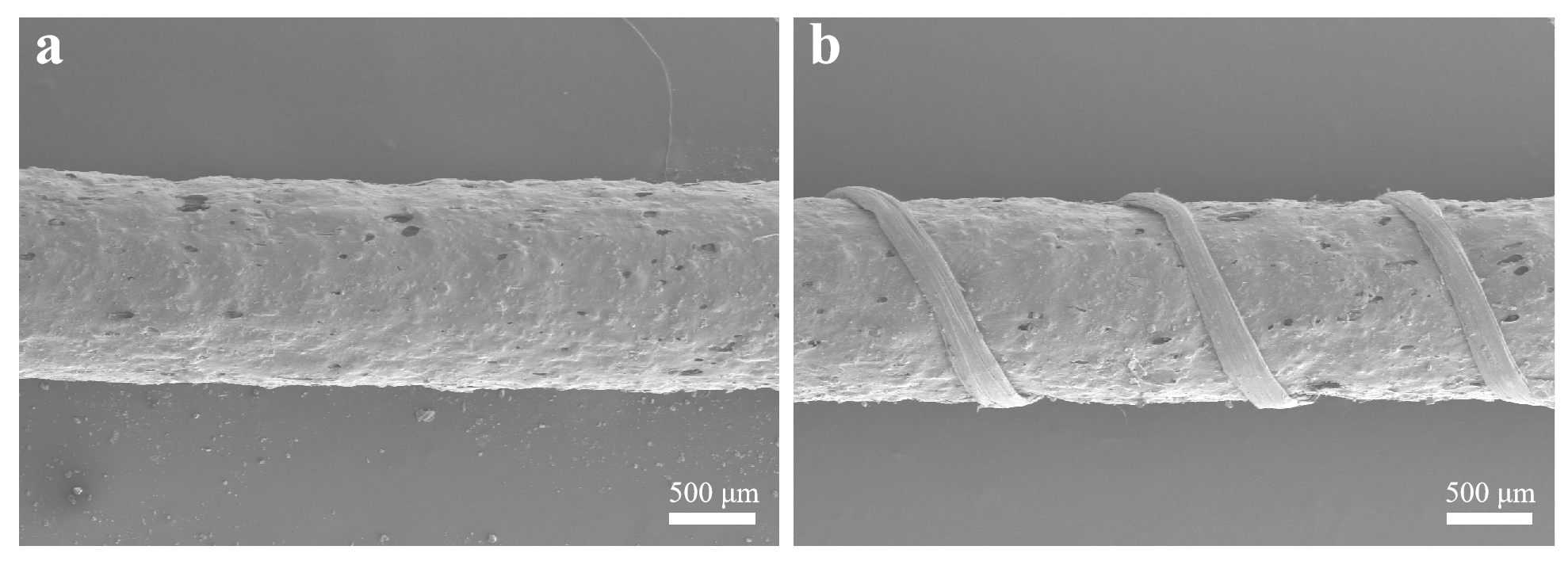


**Figure S2.** SEM images of (a) the substrate and (b) the wound fibers.


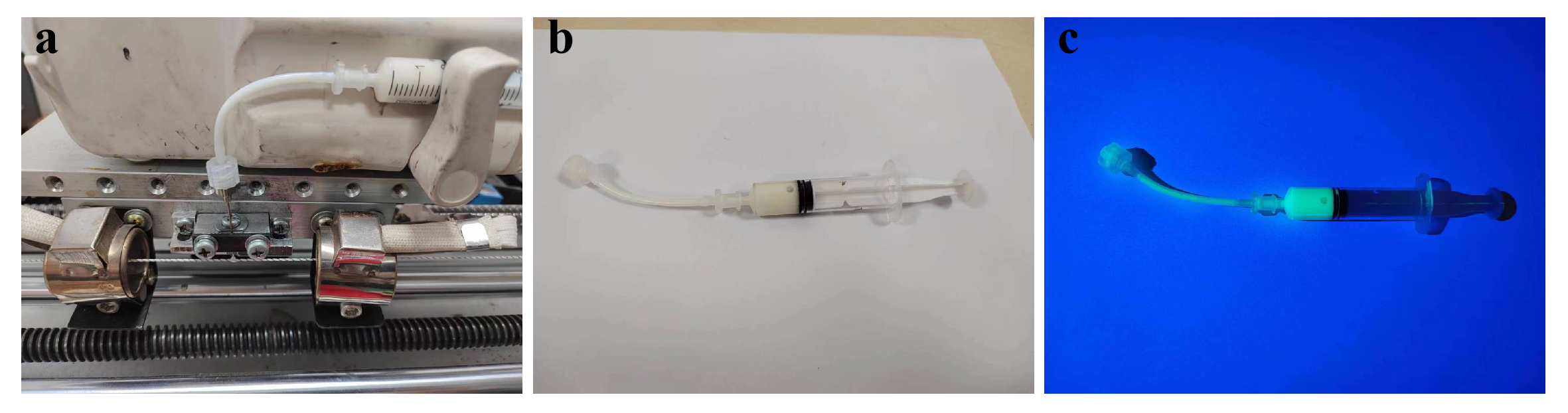


**Figure S3.** Photographs of (a) the coating apparatus and the Ecoflex/ZnS:Cu solution under (b) sunlight and (c) ultraviolet light.


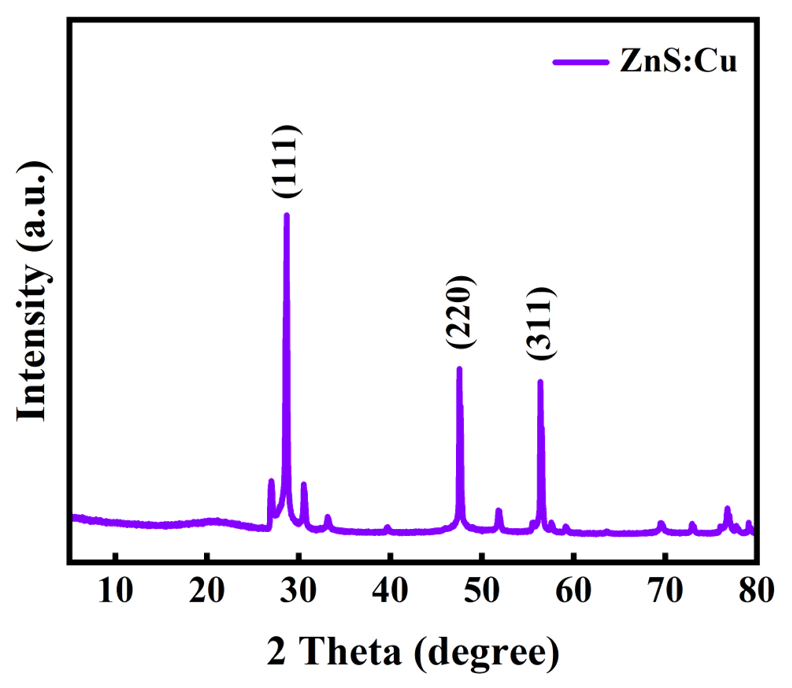


**Figure S4.** X-ray diffraction pattern of the ZnS:Cu powder sample.

**
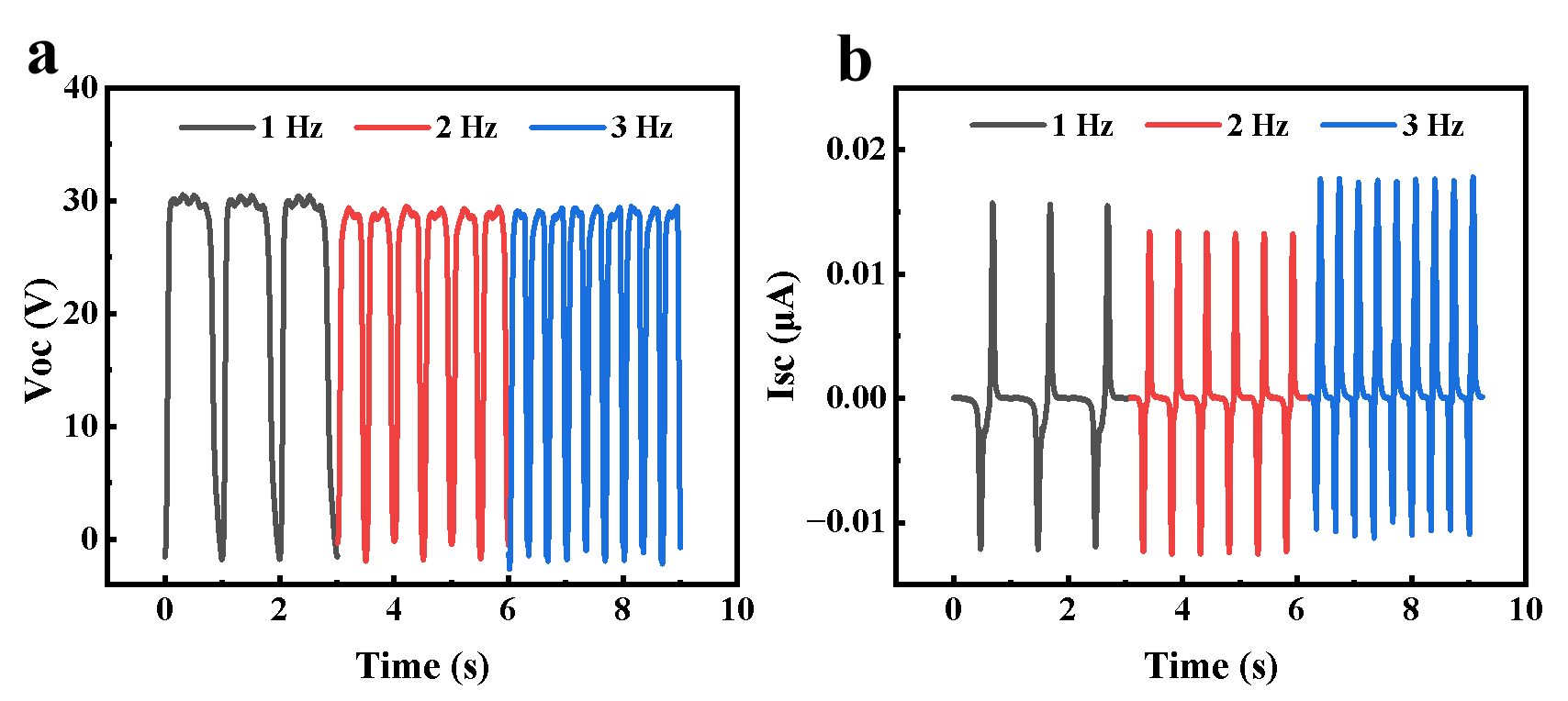
**

**Figure S5.** Under a specific stretch level (200%), Variations in (a) V_OC_ and (b) I_SC_ at different frequencies (1 to 3 Hz).


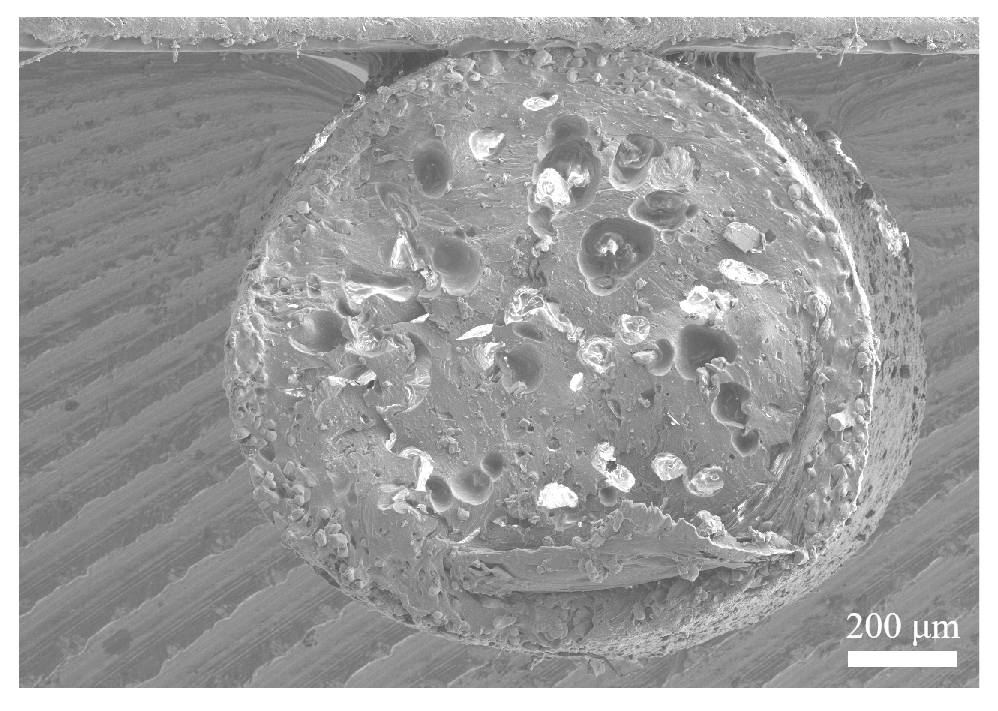


**Figure S6.** SEM image of the MLTENGF cross-section after loading cycles.


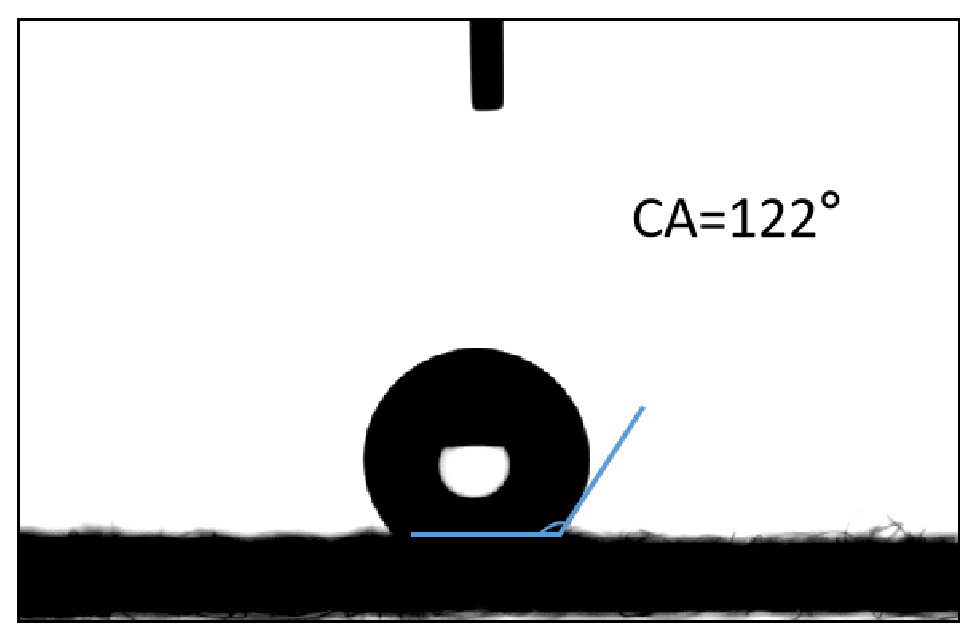


**Figure S7.** The contact angle of the MLTENGF.


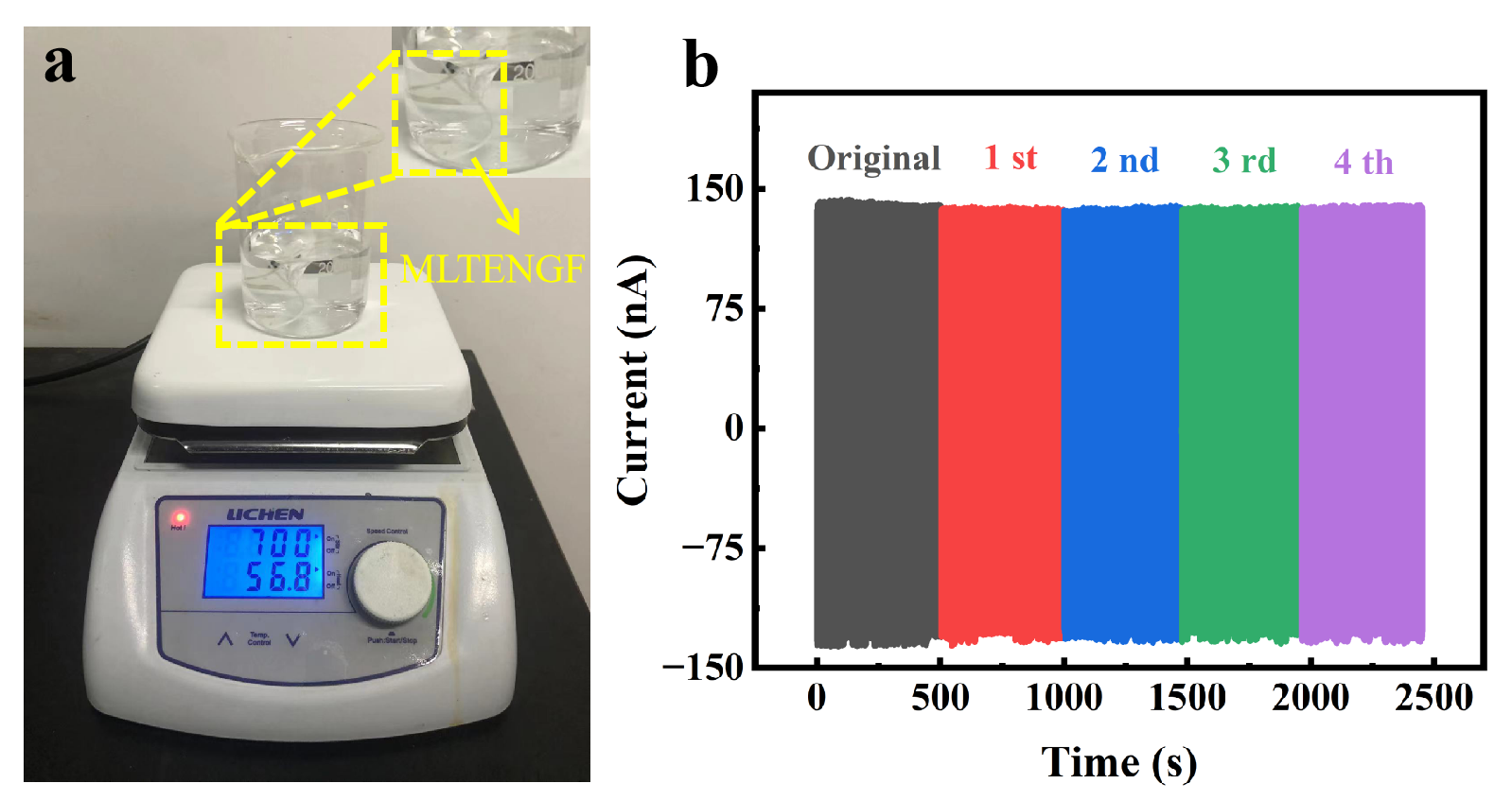


**Figure S8.** (a) Photograph of MLTENGF subjected to washing in a simulated household laundry environment using a stirring rod. (b) Under a specific stretch level (200%), Electrical current output of MLTENGF after repeated washing cycles.


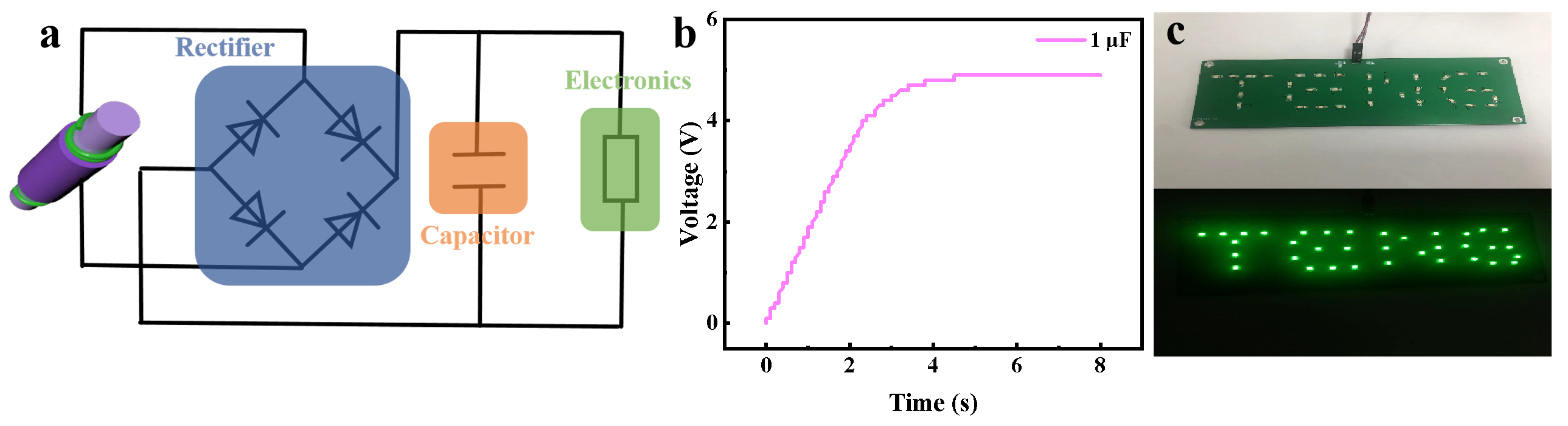


**Figure S9.** (a) Equivalent circuit diagram for charging capacitors and powering LEDs using MLTENGF. (b) Charging curve of commercial capacitors using MLTENGF. (c) Photograph demonstrating the use of MLTENGF to power LEDs displaying the word "TENG".


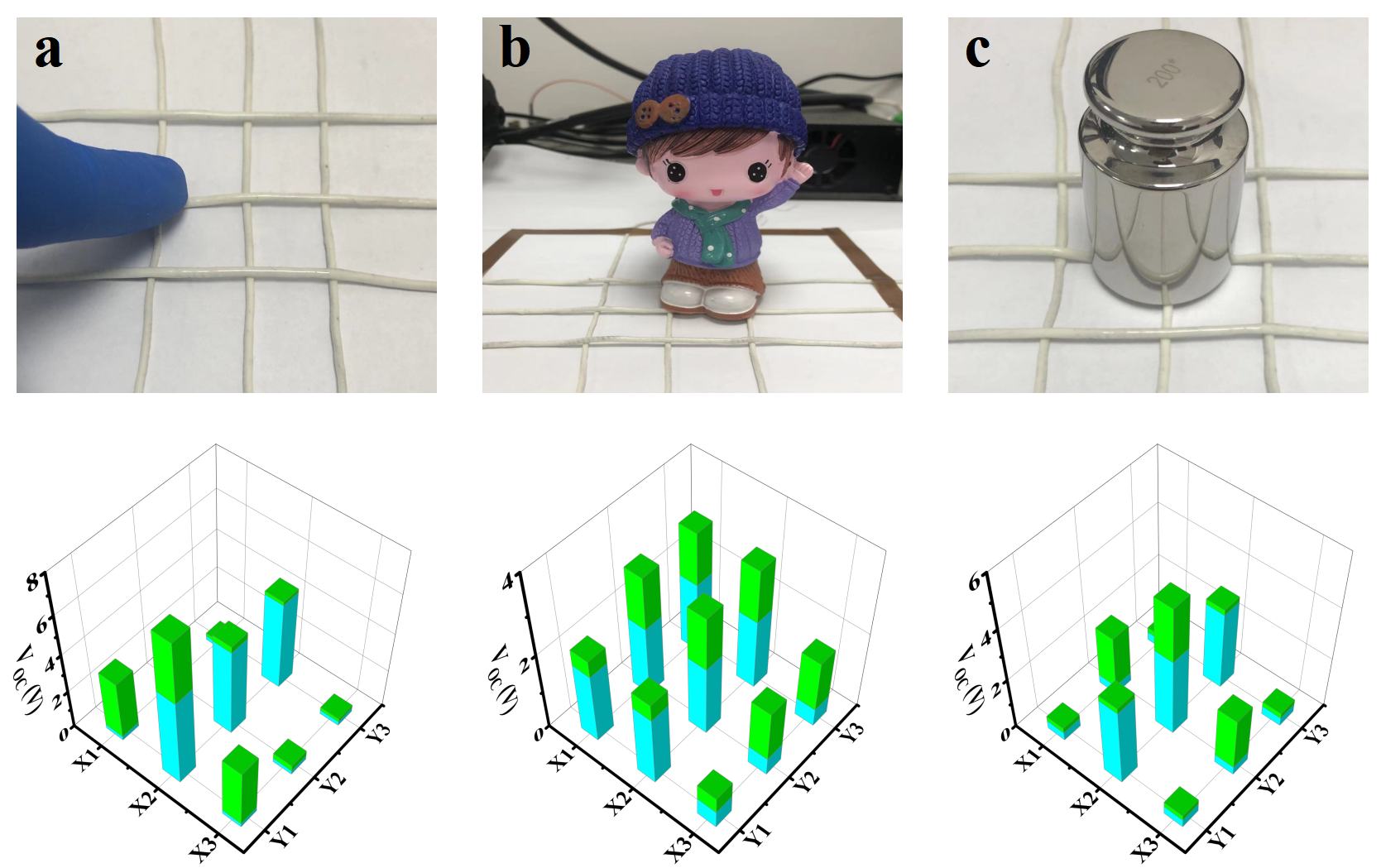


**Figure S10.** Corresponding pressure distribution maps of the stimulated MLTENGF array using (a) a finger, (b) a figurine, and (c) a 200 g weight, respectively.


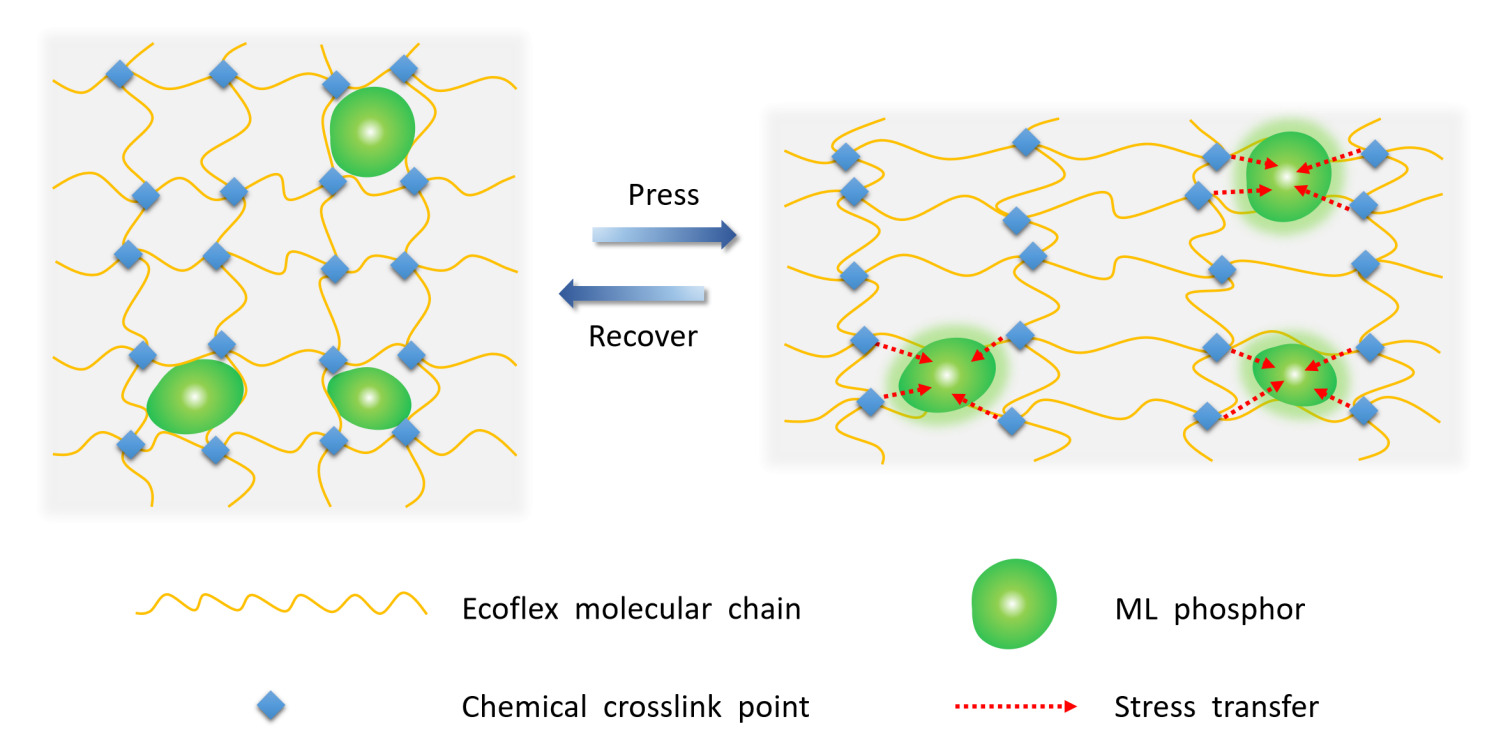


**Figure S11.** The stress transmission mechanism of chemically cross-linked thermosetting elastomer Ecoflex/ZnS:Cu composite material.


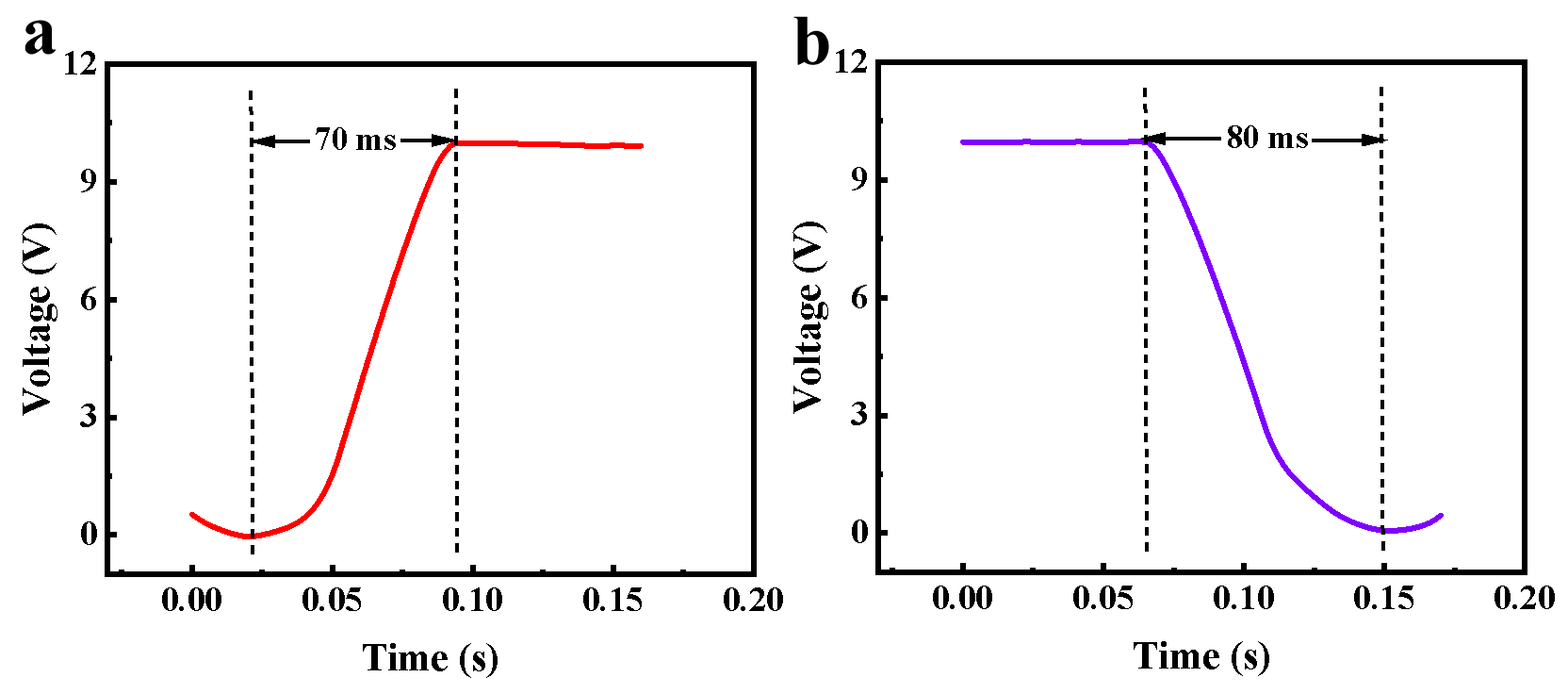


**Figure S12.** Under a specific stretch level (200%), (a) the response and (b) recovery time of MLTENGF.


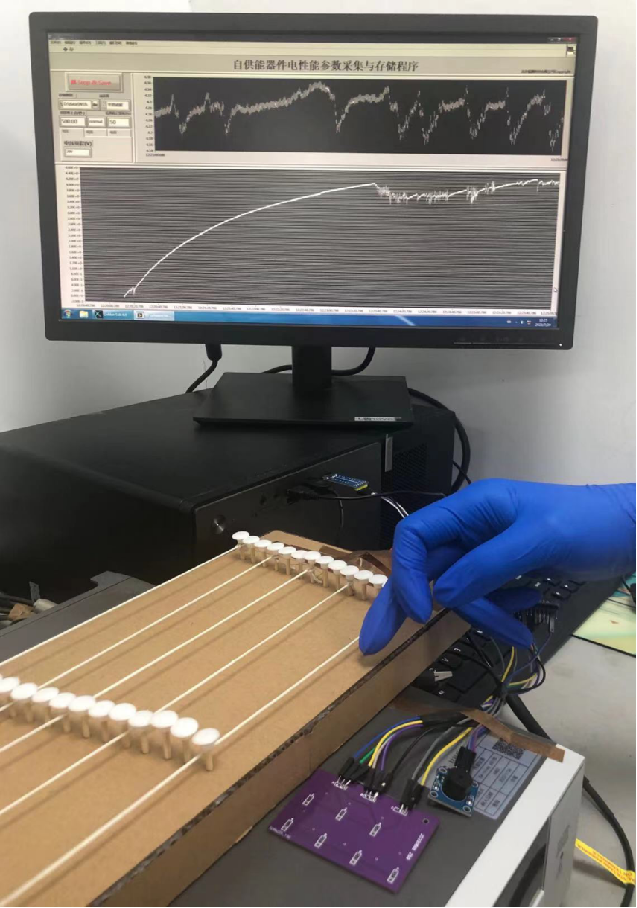


**Figure S13.** Photograph of the "intelligent zither" based on MLTENGF sensors.


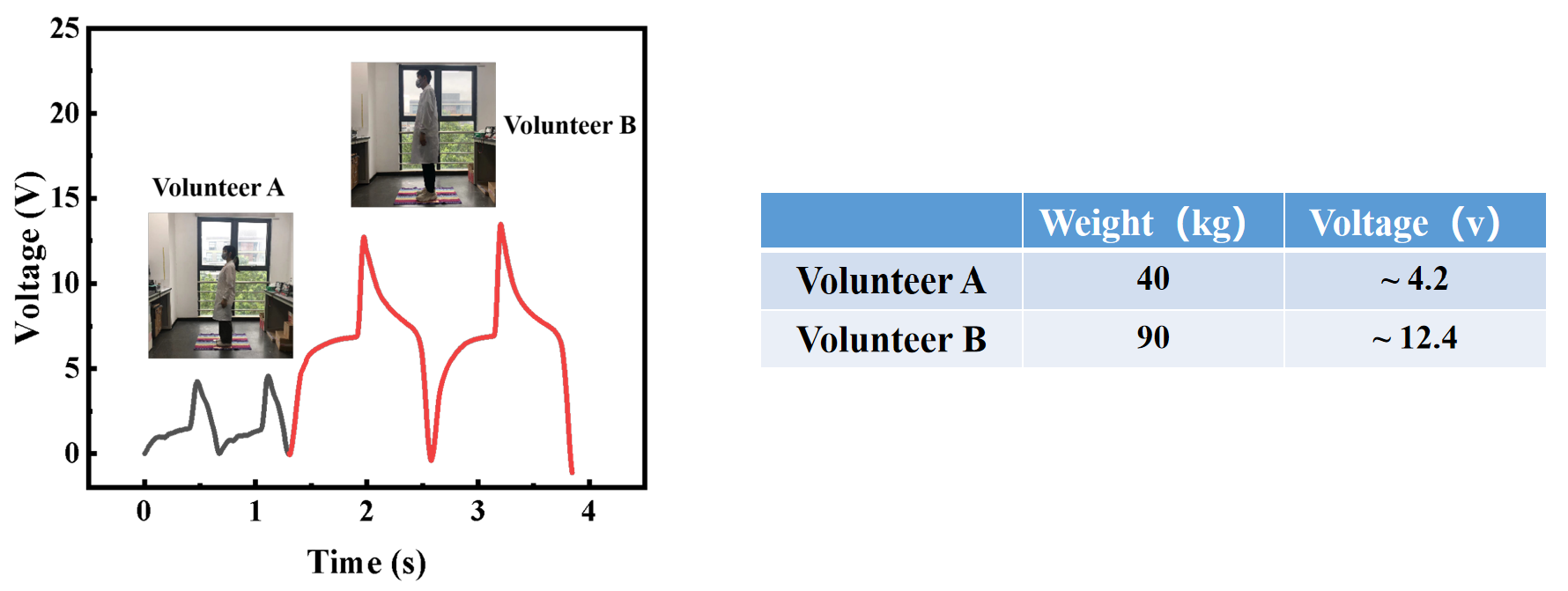


**Figure S14.** Volunteer A (weight:40 kg) and Volunteer B (weight:90 kg) stepping on the output voltage of the anti-theft alarm carpet based on MLTENGF, respectively.


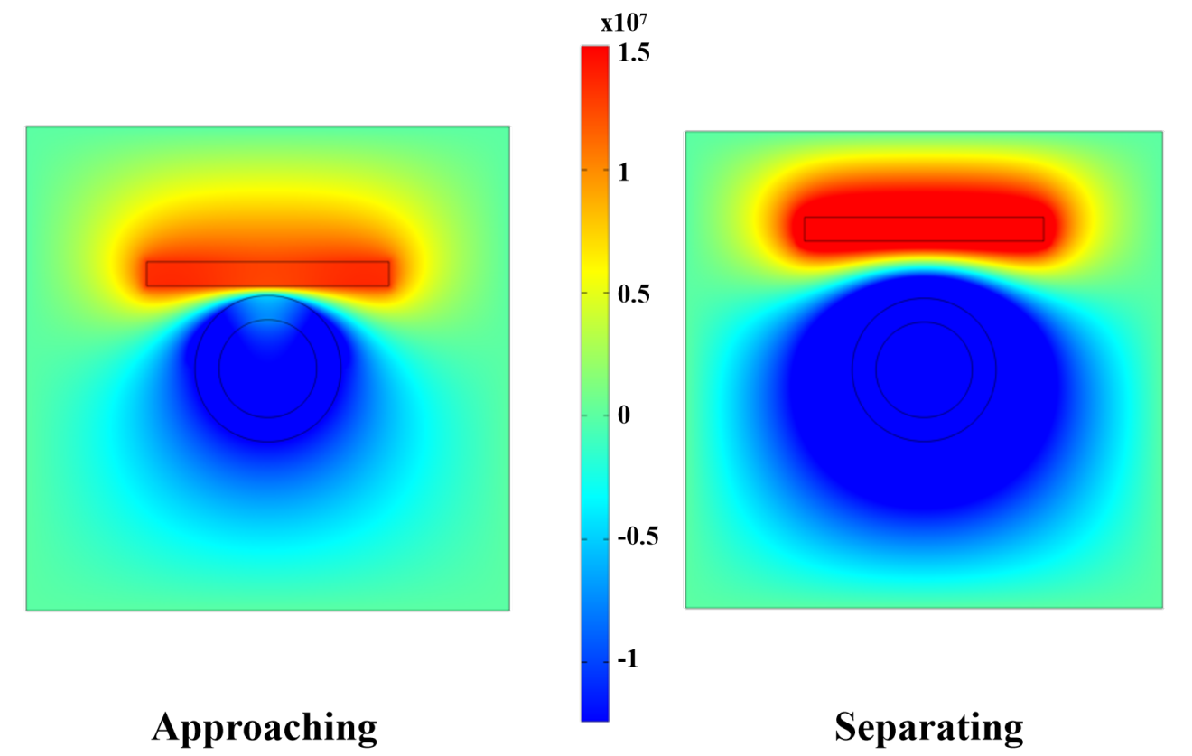


**Figure S15.** Simulated the corresponding electric potential distribution under open-circuit conditions using COMSOL software.


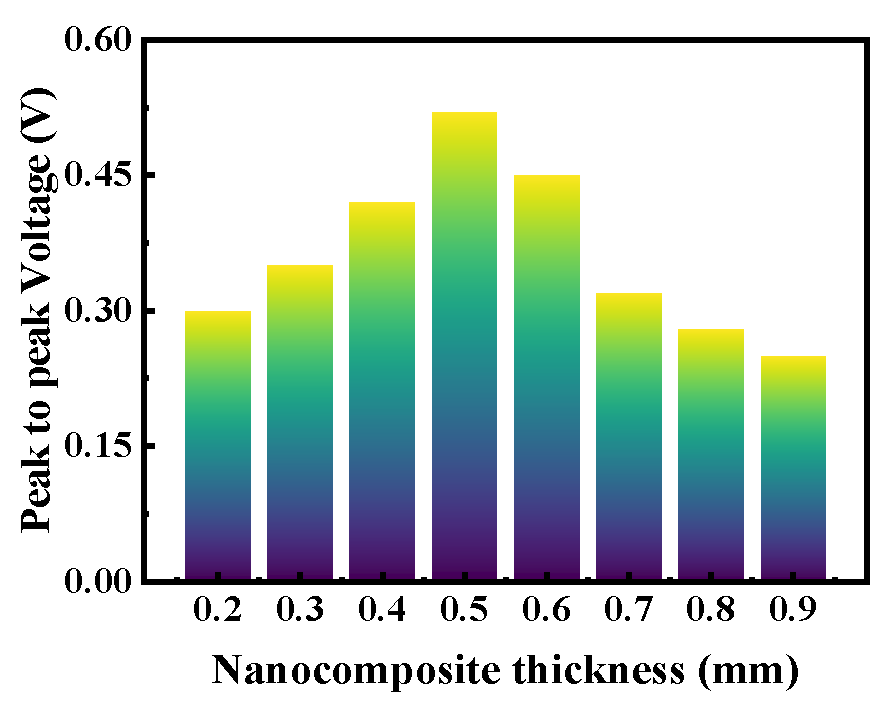


**Figure S16.** Peak voltage at different Ecoflex thicknesses.


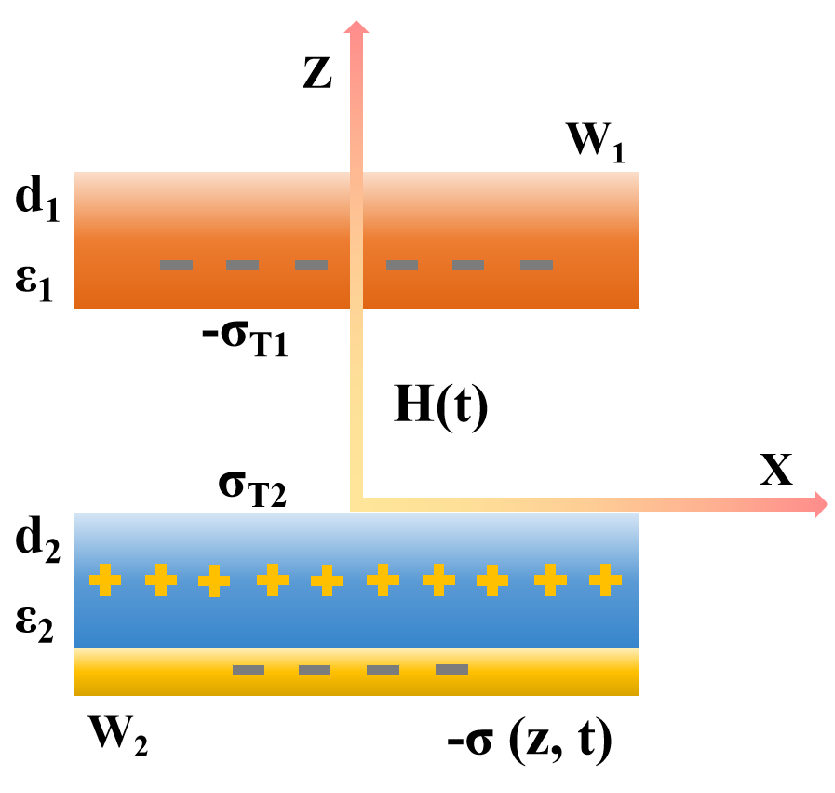


**Figure S17.** Charge distribution and parameter definitions in single-electrode mode.


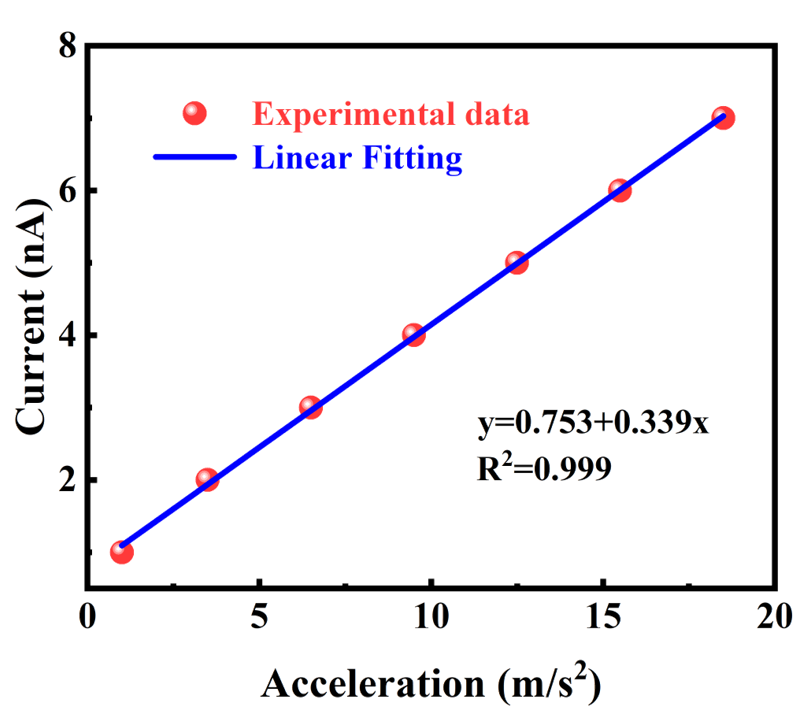


**Figure S18.** Short-circuit current and the short-circuit current response acceleration of MLTENGF in non-contact conditions.


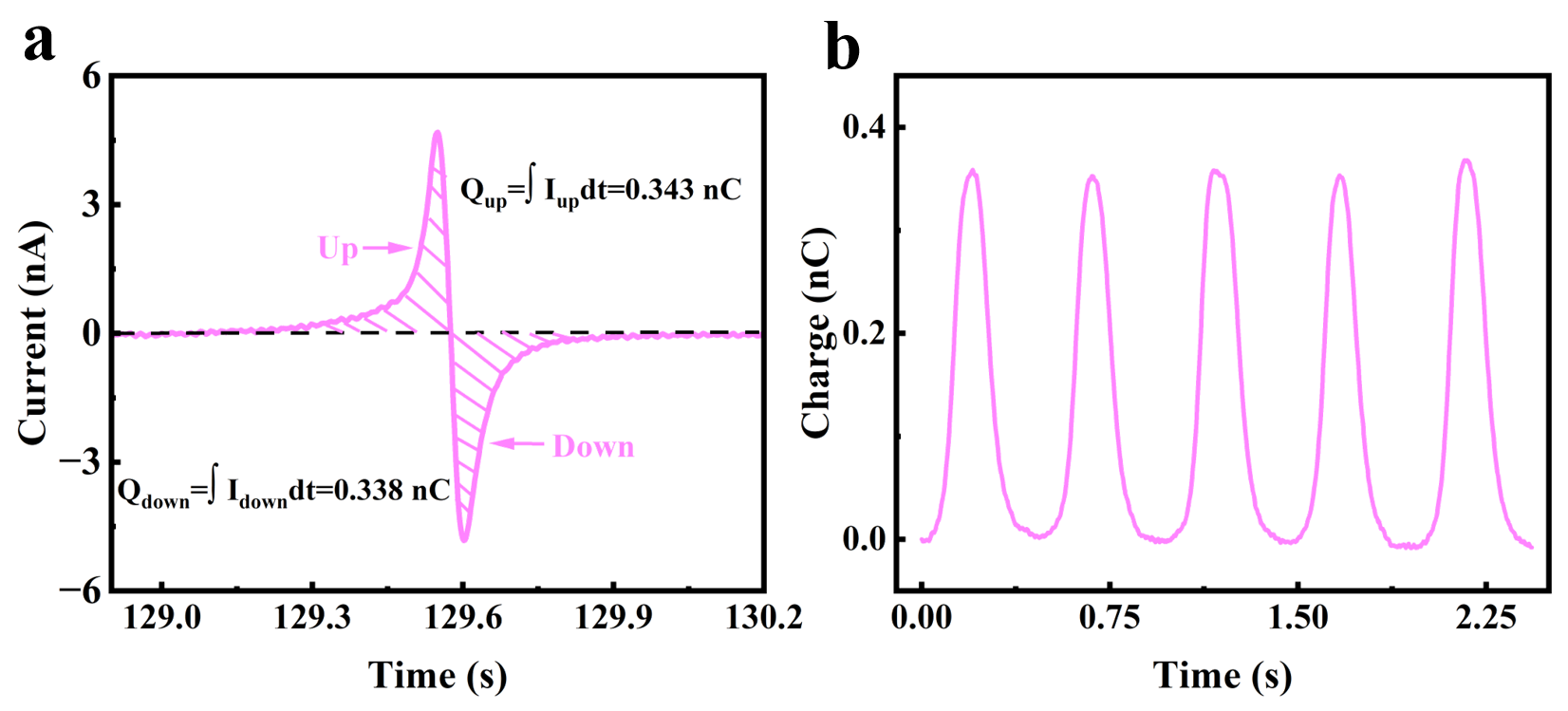


**Figure S19.** (a) Integration of the current-time curve to calculate transferred charge. (b) Measurement of transferred charge in MLTENGF using an electrostatic meter.


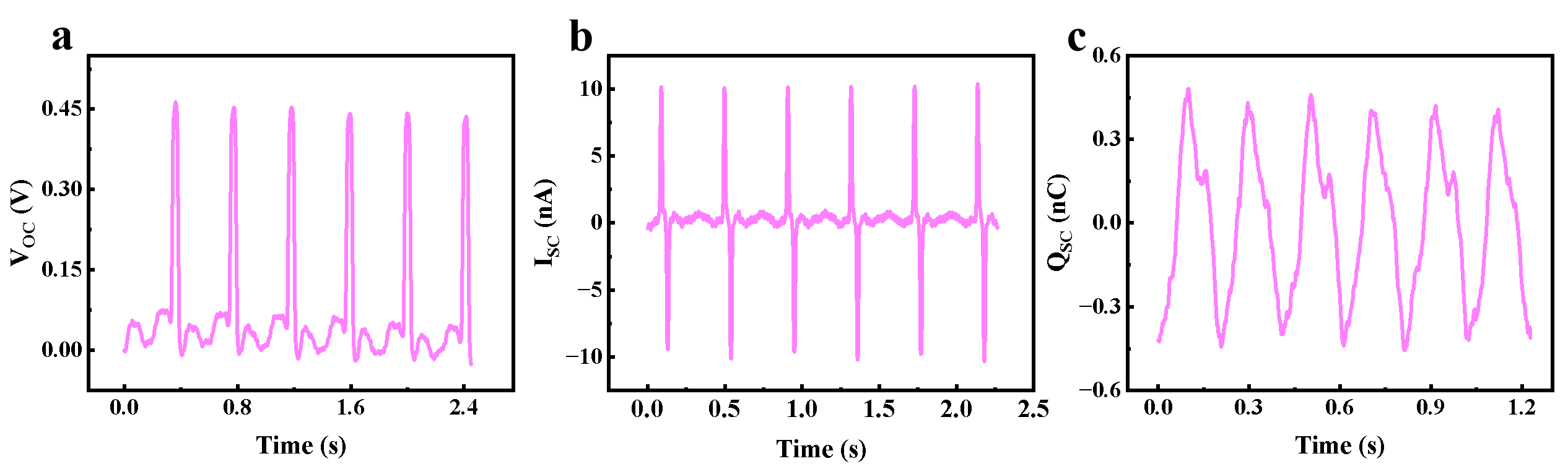


**Figure S20.** Variations in (a) V_OC_ , (b) I_SC_ , and (c) Q_SC_ under non-contact conditions.


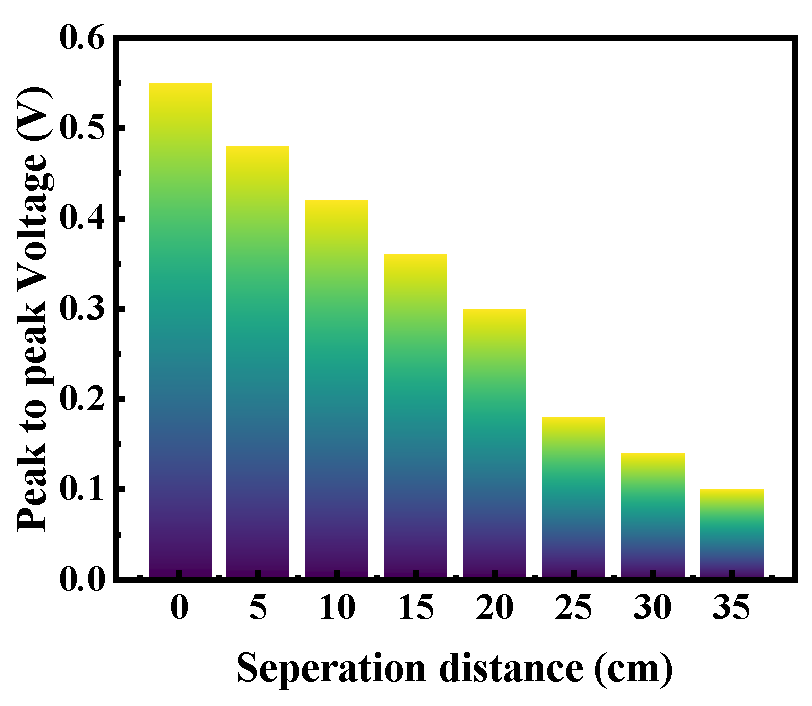


**Figure S21.** Peak voltage of MLTENGF at different separation distances.


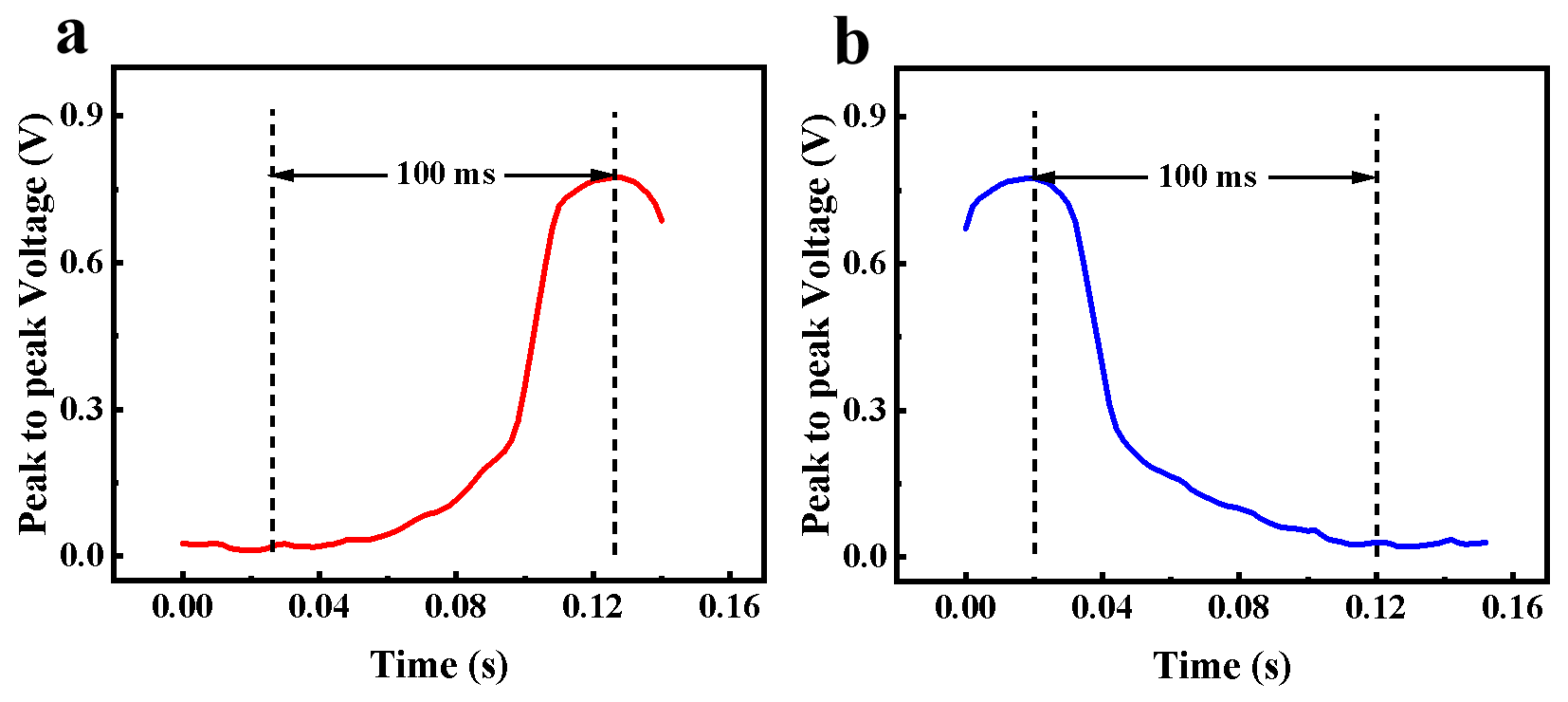


**Figure S22.** (a) The response and (b) recovery time of MLTENGF under non-contact conditions.


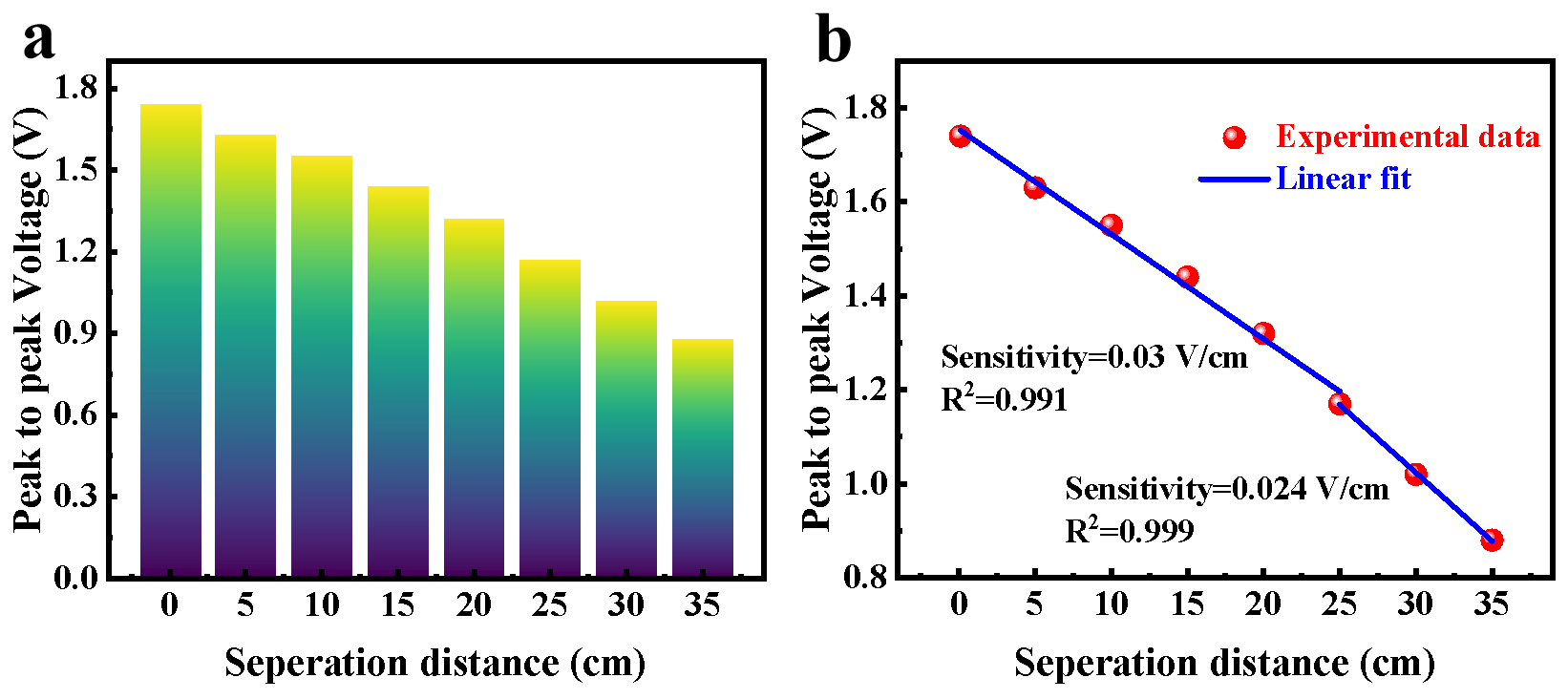


**Figure S23.** (a) Non-contact MLTENGF response to different distances and (b) sensitivities in an underwater environment.


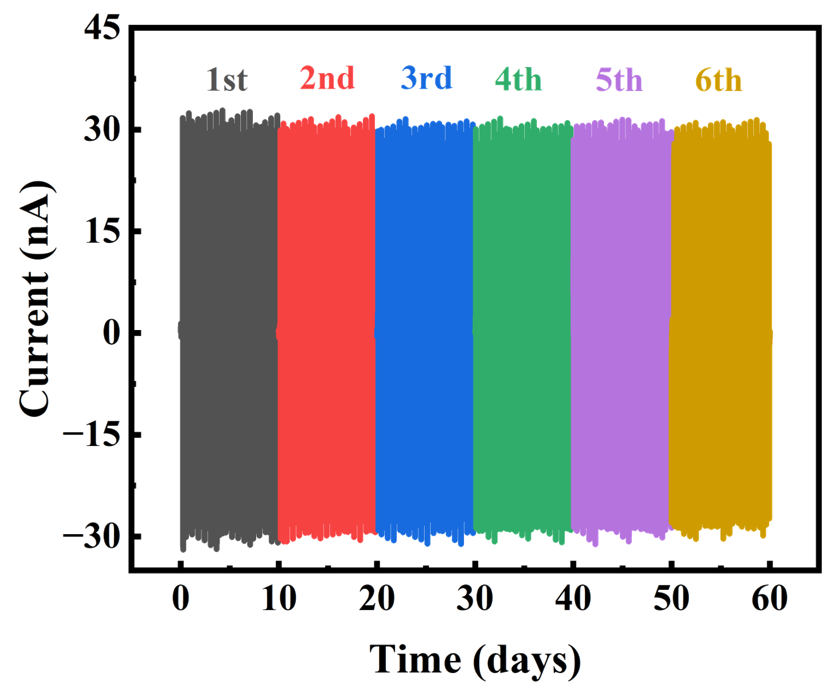


**Figure S24.** The 60 days cyclic stability of non-contact MLTENGF in an underwater environment.

**Formula Derivation**

The variation in TENG output voltage under different thicknesses of Ecoflex/ZnS:Cu composite materials is investigated. The induced surface charge density (σ) can be expressed a

|  | $\sigma=\frac{V\varepsilon_{0}\varepsilon_{r}}{d}$ | (S1) |
| --- | --- | --- |

In **Equation S1**, V represents the surface potential of Ecoflex/ZnS:Cu, ε_0_ and ε_r_ are the dielectric constants of air and the Ecoflex/ZnS:Cu composite material, and d is the thickness of the Ecoflex/ZnS:Cu composite material. To determine the optimal thickness of the composite material, TENGs with varying composite material layer thicknesses were fabricated and their output performance measured, as depicted in **Figure S15**. The output voltage increases with thicker composite material layers due to charge accumulation on the material's surface, with a maximum output voltage of 0.52 V. However, excessively thicker composite material layers may lead to reduced TENG output voltage, attributed to decreased charge density resulting from increased material resistance. Consequently, the optimal thickness was selected for further measurements. The open-circuit voltage corresponding to the single-electrode bilayer TENG is expressed as:

|  | $V_{oc}=\frac{\sigma A}{2C_{0}}$ | (2) |
| --- | --- | --- |

In the equation, σ represents the induced surface charge of the Ecoflex/ZnS:Cu composite material, A denotes the effective area between human skin and the Ecoflex/ZnS:Cu composite material layer, and C_0_ stands for the capacitance of the TENG. Notably, Voc increases with the augmentation of induced surface charge. The theoretical analysis of output current dependence on acceleration is illustrated in **Figure S16**, which shows the charge distribution and parameter definitions for describing the single-electrode mode. According to Maxwell's displacement current theory, under short-circuit conditions, the displacement current density (J_D_) equation for the TENG when short-circuited is:

|  | $J_{D}=\frac{\partial D_{Z}}{\partial t}=\frac{\partial\sigma(z,t)}{\partial t}\approx\sigma_{T}\frac{dHd_{1}\varepsilon_{0} /+d_{2}\varepsilon_{0} /}{dt{[d_{1}\varepsilon_{0} /+d_{2}\varepsilon_{0} /+z]}^{2}}$ | (3) |
| --- | --- | --- |

In **Equation 3** where ε_1_ and ε_2_ represent the dielectric constants, d_1_ and d_2_ are the thicknesses, σ_t_(t) is the frictional charge density, σ(z, t) is the surface free electron density of the electrode layer, and H is a function of time (t) determined by the contact/separation rate between the two dielectrics. The magnitude of the displacement current is directly proportional to the contact/separation velocity (dH/dt) between the two dielectrics. We define the acceleration applied to the dielectric without attached electrodes as 'a.' The displacement current density can be expressed as:

|  | $J_{D}\approx\sigma_{T}\int_{0}^{t}a(t)dt\frac{d_{1}\varepsilon_{0} /+d_{2}\varepsilon_{0} /}{{[d_{1}\varepsilon_{0} /+d_{2}\varepsilon_{0} /+z]}^{2}}$ | (4) |
| --- | --- | --- |

In **Equation 4**, it can be observed that the magnitude of the displacement current is directly proportional to the acceleration (a). Fitting the short-circuit current and acceleration data reveals a strong linear relationship between the two, with a correlation coefficient (R^2^) of 0.999. Therefore, in the experiments involving TENG-based acceleration sensors, a higher acceleration results in a greater output current, as depicted in **Figure S18**. The integral calculation of the current-time curve for charge transfer yields a value of 0.34 nC, which is consistent with the experimentally measured transfer charge of 0.35 nC obtained using an electrostatic meter (**Figure S19a-b**). TENG voltage, current, and charge signals triggered by hand at a fixed frequency are shown in **Figure S20a-c**. As per **Equation 2**, it is evident that the peak-to-peak voltage is influenced by the separation distance. At a separation distance of 0.1 cm, the maximum peak-to-peak voltage is 0.55 V, and the TENG sensor exhibits two linear regions, R1 and R2. In the R1 region (0.1 ~ 25 cm), the sensitivity is 0.015 V cm^-1^ with a linear fit of 0.982; in the R2 region (25 ~ 35 cm), the sensitivity is 0.013 V cm^-1^ with a linear fit of 0.999. The distance detection threshold spans from 0.1 cm to 35 cm, where the voltage decreases from 0.55 V to 0.1 V. The peak voltage decreases as the separation distance increases (**Figure S21**). This property is likely due to the decreasing electric field with an increasing separation distance.
